# Road Map of Semiconductor Metal-Oxide-Based Sensors: A Review

**DOI:** 10.3390/s23156849

**Published:** 2023-08-01

**Authors:** Taposhree Dutta, Tanzila Noushin, Shawana Tabassum, Satyendra K. Mishra

**Affiliations:** 1 Department of Chemistry, IIEST Shibpur, Howrah 711103, West Bengal, India; taposhreedutta812@gmail.com; 2 Department of Electrical and Computer Engineering, The University of Texas at Dallas, Richardson, TX 75080, USA; tanzila.noushin@utdallas.edu; 3 Department of Electrical Engineering, The University of Texas at Tyler, Tyler, TX 75799, USA; stabassum@uttyler.edu; 4 Danish Offshore Technology Center, Technical University of Denmark, 2800 Lyngby, Denmark; 5 SRCOM, Centre Technologic de Telecomunicacions de Catalunya, 08860 Castelldefels, Barcelona, Spain

**Keywords:** semiconductor metal oxides, gas sensor, chemical sensor, biosensor, conduction band, valence band, p-n junction

## Abstract

Identifying disease biomarkers and detecting hazardous, explosive, flammable, and polluting gases and chemicals with extremely sensitive and selective sensor devices remains a challenging and time-consuming research challenge. Due to their exceptional characteristics, semiconducting metal oxides (SMOxs) have received a lot of attention in terms of the development of various types of sensors in recent years. The key performance indicators of SMOx-based sensors are their sensitivity, selectivity, recovery time, and steady response over time. SMOx-based sensors are discussed in this review based on their different properties. Surface properties of the functional material, such as its (nano)structure, morphology, and crystallinity, greatly influence sensor performance. A few examples of the complicated and poorly understood processes involved in SMOx sensing systems are adsorption and chemisorption, charge transfers, and oxygen migration. The future prospects of SMOx-based gas sensors, chemical sensors, and biological sensors are also discussed.

## 1. Introduction

In sensing applications, metal oxides (MOxs (metal oxides), mainly II–VI semiconductors) are mostly used due to their inexpensiveness, ease of manufacture, quick response time, wide detection range, and resistance to harsh conditions [1,2]. For sensors to be effective, (i) there must be a charge transfer between the analytes and sensing materials and (ii) the measurement must have an analyte concentration dependence [3,4]. In addition, there is a need for efficient and effective methods for detecting volatile, chemical, and biological compounds and molecules. Analytical chemistry methods, such as spectrophotometry, fluorometry, gas chromatography (GC), and high-performance liquid chromatography (HPLC), were previously used to detect these molecules accurately. Furthermore, these methods were heavy, expensive, had low throughput, time-consuming pretreatment steps, required highly skilled operators and significant power consumption, and did not provide real-time information for risk reduction or decision-making [5]. As a result of these limitations, most present sensing methods rely on SMOx-based materials for sensing applications, such as 1D and 2D field effect transistors (FET) and the Internet of things (IoT) [3,4,6,7,8].

Compounds with high levels of ionic bonding or electrostatic interaction are called semiconductor metal oxides (SMOxs) [9,10]. In recent years, they have attracted a great deal of attention due to their excellent sensing capabilities, adaptability in terms of size, ease of manufacture, and low power consumption. By tuning the size and composition of the materials, the electrical, optical, mechanical, catalytic, and magnetic properties can be modulated. Because of their large surface-area-to-volume ratio, SMOxs, which have a size range between 1 and 100 nm, exhibit unique physical and chemical properties [11,12,13,14,15,16]. In addition to the size and geometry of the SMOx material, electron transport is also influenced by it [17,18]. Adding dopants, impurities, composite structures, or metal additives can further manipulate the charge transfer in a SMOx. In addition to improving the long-term stability and selectivity, these components lower the operation temperature, energy consumption, and humidity interference of pristine SMOx [19,20,21,22,23]. There are two types of SMOx materials: n-type and p-type, depending on the type of dopants. In SMOx, oxygen vacancies serve as electron donors, and hence, these compounds are n-type. Conversely, p-type SMOx, such as CuO, Co_3_O_4_, Cr_2_O_3_, and NiO, have metal ions and are electron acceptors. As a result of their tunable sensitivity, selectivity, and response time, SMOx-based materials are widely used in sensors that detect gases, chemicals, and biomolecules. With SMOx-based sensors, a wide range of gases can be detected, such as oxidizing gases and reducing gases [17,23].

Here, we present a brief review of the fundamentals and sensing properties of SMOx materials; the factors that influence their sensing performance; and their applications in gas, chemical, and biological sensing. There was a variety of literature available on SMOx gas sensors [24,25,26,27,28,29,30,31] and biosensors [32]. This article reviewed different aspects of SMOx materials and their sensing properties in one comprehensive article. This review provides researchers with a better understanding of the fundamentals and sensing applications of SMOx, enabling them to develop the next generation of SMOx-based sensors.

## 2. Fundamentals of Semiconductor Metal Oxides

The easy charge transfer properties of metal oxides (MOxs) make them unique among semiconducting materials. This effect is due to the large electronegativity difference, and thus, the high degree of ionic bonding, between the metal and oxygen that the MOx has. MOx has a conduction band minimum (CBM) and valence band maximum (VBM) of metal (M) ns and oxygen (O) 2p orbitals, respectively. Metals (Ms) and oxygen (O) have highly dispersed or localized orbitals (ns and 2p). Furthermore, metal oxides have a much higher dispersive valence band maximum (VBM) than n-type semiconductors. As an example, In_2_O_3_, ZnO, SnO_2_, and their hybrid composites function as n-type MOx, while NiO and Cu_2_O are p-type MOx. The first-known p-type transparent conductive oxide (TCO) was nickel oxide (NiO) [33].

The mobility (μ) of a carrier is inversely proportional to its effective mass (m*) and is given by the following equation:

μ = e τ/m*

where τ is the free carrier scattering time

Controlled physical and chemical properties of metal oxides, including structural defects, morphology, grain size, and specific surface area, enable them to be used in a range of applications, such as catalysis, sensors, energy conversion, and environmental monitoring [34,35].

## 3. Properties of Semiconductor-Metal-Oxide-Based Sensors

Regarding the greenhouse effect, MOx-based sensors are used for the rapid detection of harmful and toxic gases, where the low concentration (in ppm or ppb) of the target gas is converted into a measurable electrical, optical, or magnetic signal. In these sensors, metal oxide semiconductors and metal oxide–polymer composites are used to produce excellent sensitivity [36,37,38]. As a result, semiconducting metal oxides (SMOxs) can be used to detect low gas concentrations with a high sensitivity and rapid response. A SMOx-based sensor is characterized by its low cost, rapid response and recovery time, high stability, simple electronic interface, and low maintenance, making it an ideal and promising material for detecting toxic gases [5,31,39,40]. Materials made of SMOx are ionic solids, which are held together by strong ionic bonds between positive metallic and negative oxygen ions. Semiconductor metal oxides (SMOxs) have filled electronic shells, making them more thermally and chemically stable than free metal oxides. Incomplete electronic shells d endow optical properties, such as high dielectric constants [39,41,42,43]. Depending on the design, a SMOx can be flexible; porous; and can be in a zero-dimensional shape (0D), a 1D shape, a 2D shape, or a 3D shape [44,45,46]. As the temperature increases, SMOx materials’ conductivity (and hence resistance) changes. Moreover, optical, electrical, and magnetic fields affect the conductivity of SMOx.

Understanding semiconductor metal oxides (SMOxs) is crucial to developing sensors with high sensitivity. SMOx sensing properties are affected by physical factors, such as crystalline structure, defects, energy bands, impurities, charge transport, and p-n junction formation. In a SMOx, charge transfer can be controlled by doping with donor materials [47,48,49].

### 3.1. Crystalline Structure with Defects

Due to noble metal doping, materials with high crystalline structures have been investigated to develop sensors. SMOx can be classified into two types of crystalline structure: monocrystalline and polycrystalline. A monocrystalline structure is formed by a regular arrangement of atoms. Conversely, polycrystalline structures consist of small single crystals arranged randomly. On the other hand, non-crystals possess irregular shapes with short-range structural order [50,51,52]. As an example, the surface of a crystal of SnO_2_ usually lacks one or more atoms, resulting in abundant unsaturated bonds. Consequently, SnO_2_ exhibits high chemical activity and participates in redox reactions [53,54]. SnO_2_ has a tetragonal crystal structure [55,56], while ZnO has a hexagonal structure [44,57]. Semiconductor metal oxides (SMOxs) have special crystal structures that influence their physiochemical activity (Table 1).

There are various types of defects in SMOx, including point defects, line defects, plane defects, and volume defects. SMOx’s physio-chemical activity can be enhanced by partial defects caused by impurities [101,102]. Photoelectric activity can induce point defects, also called 0D defects [103,104]. Another type of defect is a line defect, which is caused by partial crystal slides. There are two types of dislocation defects: closed rings and surface defects. There are also planar defects, which can include angular grain boundaries, stack layer faults, and twin crystals. In the crystal matrix, volume defects are voids with different structures, densities, and chemical compositions [105,106].

### 3.2. Energy Band of SMOx in the Presence of Impurities

**Figure 1:**
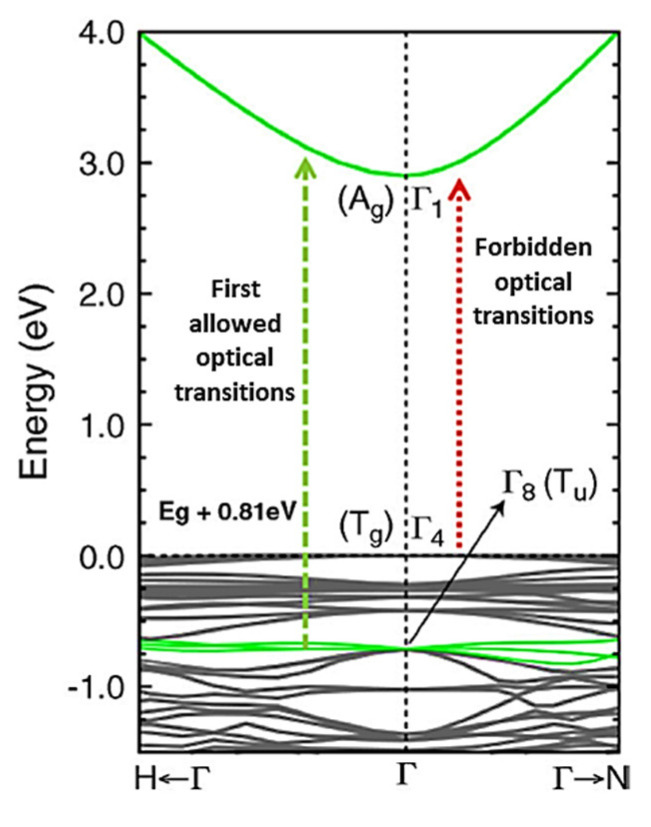
Band structure of In_2_O_3_ near the Brillouin zone. Here, a weak optical absorption is observed at 2.7 eV and a strong optical transition occurs between lower − lying valence bands [112].

In addition, the sensing property of n-type semiconductors and p-type semiconductors depends on the energy band structure of semiconductor metal oxides (SMOxs). When the SMOx thickness reaches a level comparable to the depletion layer width, the energy band is no longer constrained to the surface but is affected by a significant number of grains, which, in turn, affects the electronic structure and electron–hole charge carriers [107,108,109]. In general, an electron’s conduction energy band becomes vacant when the minimum band gap energy (Eg) of the SMOx is reached (Figure 1). As a result, the valence band is left with holes. Electrons (e^−^) and holes (h^+^) are easily mobilized in the presence of an external electric field, while at low energy, electron–hole pairs (e^−^ + h^+^) are electrostatically bound [110,111]. Since In_2_O_3_ has a small effective mass of electrons, its band structure shows a highly dispersive CBM. Its optical bandgap is 3.7 eV [112]. The presence of impurities induces intra-band electron transitions, such as electrons moving from defect states to ground states. By adjusting the size, shape, and composition of impurities, intraband gaps can be modulated. A SMOx sensor with a large Eg can work at high temperatures, which indicates that SMOx sensors are thermally stable. When the operating temperature exceeds 300 °C, gas sensors should have a band gap greater than 2.5 eV. SMOx-based gas sensors have a weakly dependent chemical activity on ambient humidity [111,113,114] (Figure 1). In some core–shell semiconductors, for instance, the conduction and valence bands of the core and shell are staggered, and electrons and holes are separated. It was found that the conduction band energy was the lowest in the shell and highest in the core. The energy band offsets in semiconductor materials segregate electrons from the shell and holes from the core, allowing carrier recombination across the interface at a lower energy than any of their constituent band gaps [110,114]. Electron-saturation velocities are high, heterojunctions are readily available, gaps are broad, and breakdown fields are large, allowing for fast and very sensitive gas detection systems to operate. The size and shape of semiconductor materials can be controlled by applying strain due to quantum confinement phenomena. It is possible to adjust the bandgap range of semiconducting nanostructures due to their high elastic limit. The band structure governs the adsorption of light, charge separation, and recombination of charge, which determines the use of a SMOx in photoelectric conversion [109,114]. 

Different electron–hole carriers produced by doped SMOx affect the conductivity [108,115]. There are two types of doped semiconductors: n-type and p-type [116,117,118]. The doping of materials regulates their conductivity and mass transfer, which is extremely important for gas sensors.

### 3.3. Carrier Transportation and Electronic Structure of SMOx

Conductivity in semiconductor metal oxides is affected by the production of free carriers, e.g., electrons in n-type semiconductors and holes in p-type semiconductors [119,120,121]. A stable concentration of conductive electrons and holes is maintained at thermal equilibrium [50,106,108]. Metal oxide’s electronic structure, temperature, applied electronic field, doping, and lack of structural order in the material can influence carrier transport mechanisms, such as drift, diffusion, and recombination [122,123]. The movement of carriers from a high concentration to a low concentration is called diffusion [124,125]. Also, the carrier recombination rate affects the carrier lifetime and gas-sensing properties [110,126].

Its d valence bond orbitals impart unique physical and chemical properties for various applications, such as gas sensing [29,46].

### 3.4. Formation of p-n Junctions in a SMOx

**Figure 2:**
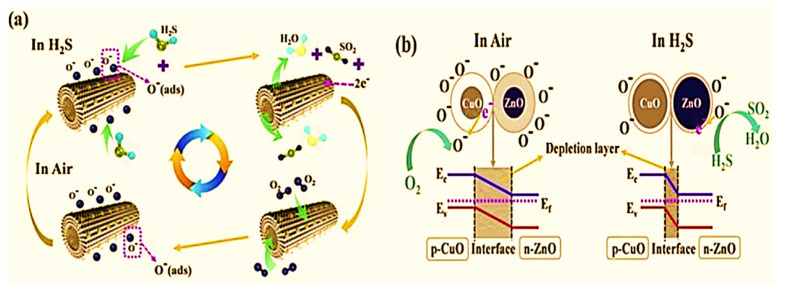
Schematic illustration of (**a**) H_2_S gas detection via hollow nanofibers and (**b**) the formation of the depletion layer at the p−n interface [130].

Generally, p-n junctions form between semiconductors with different electronic structures [127,128]. A hole diffuses from a p-type semiconductor to a n-type semiconductor, leaving negatively charged ions on the p-type semiconductor. The n-type semiconductor, however, loses free electrons, leaving positively charged ions. p-n junctions possess unidirectional conductivity since the ions cannot diffuse and form a zone of space charge at the interface. A p-n junction affects the electronic, optical, and magnetic properties of SMOx materials [103,129]. It is possible to control the properties of SMOx to design it for a wide range of applications, such as gas sensing, catalysis, and energy storage. C. Han and co-workers presented hollow nanofibers based on p-CuO/n-ZnO for gas sensing [130]. Using atomic layer deposition (ALD), electrospun heterostructures were fabricated to investigate the effect of the composition on gas sensing. As the concentration is increased, the response rate slowly decreases, with Rzn/cu = 15.6. Compared with pure ZnO and pure CuO, these heterostructures exhibit 6 and 45 times higher responses to H_2_S gas, respectively (Figure 2).

**Figure 3:**
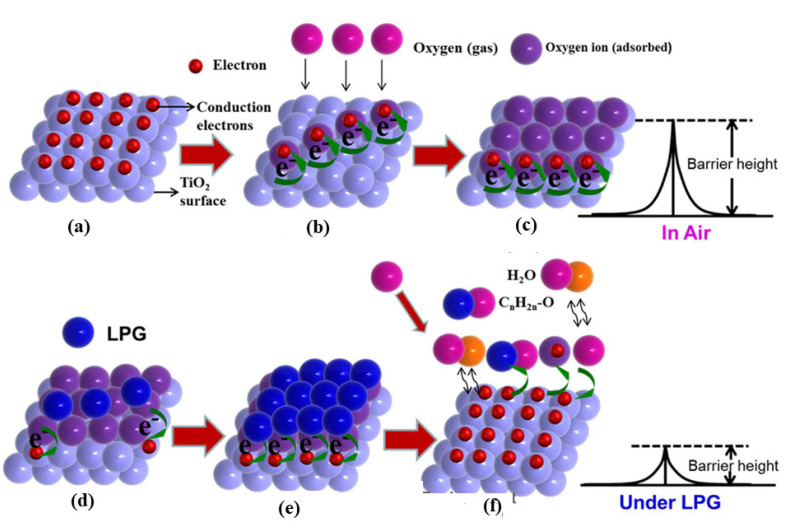
TiO_2_ sensing mechanism shown schematically with exposure to LPG and in air. (**a**–**c**) TiO_2_ sensor in air, where ionic species (O_2_^−^, O^−^ and O^2−^) form due to adsorption of oxygen from ambient air on the surface film and capture the electrons from n-type TiO_2_; (**d**–**f**) When LPG exposed and interacted with adsorbed oxygen, large number of electrons re-injected on TiO_2_ surface and decreased the barrier hight [131].

According to Dhawale and co-workers, LPG detectability changes based on resistance or barrier height with a transition metal oxide [131]. The amount of chemisorbed oxygen on the surface; charges on the surface; diffusion; and other processes, such as gas adsorption and desorption, control the electrical resistance or barrier height (Figure 3). By adsorbing oxygen from the surrounding air on the film surfaces, ionic species like O_2_-(ads), O-(ads), and O_2_-(ads) are formed. Ionic species trap electrons from the valence band (topmost) and remove them from films. In consequence, these adsorbed oxygen species reduce the conductivity of n-type TiO_2_. Whenever the chemisorption equilibrium is upset, the resistance or the barrier height of TiO_2_ changes [132]. Since LPG is a reducing gas containing components such as CH_4_, C_3_H_8_, and C_4_H_10_, gas-sensing mechanisms for LPG become more complicated [133]. During the exposure of LPG gas to a TiO_2_ sensor, chemisorbed oxygen releases trapped electrons back onto the TiO_2_ surface, resulting in a drastic reduction in the electrical resistance and barrier height. It is possible to increase the gas response by adding noble metals to metal oxide surfaces [134]. Painting Pd nanoparticles on TiO_2_ improves the response of LPG over that of pristine TiO_2_. The surface energy changes when Pd is added to TiO_2,_ and a spillover effect occurs [135]. Because of the weak interaction between the Pd atom and the oxygen gas, the Pd:TiO_2_ sensor requires a relatively low temperature to dissolve [136]. However, a remarkably significant number of electrons are injected back into the topmost conduction band of TiO_2_, thereby increasing the conductivity. Active Pd nanoparticle catalysts improve the LPG response by speeding up the process and providing more active sites. Furthermore, Lee and co-workers designed a hollow cube nanostructure with ZnO and CuO cores for acetone sensing, using the CuO (a p-type material) as a catalyst [134]. The n-type ZnO and p-type CuO domains produced a consistent p-n junction. When the two materials were still connected, charge conduction took place across the p-n junction, resulting in a balance in Fermi energies. The result was the formation of charge depletion layers and a potential barrier at the contact. Finally, the ZnO–CuO core–hollow cube nanostructures at 200 °C displayed a 680 k resistance compared with the 8.8 kΩ of the CuO hollow cubes at the p-n junction. Surface-adsorbed oxygen species were consumed and the surface charge in CuO domains was reduced by acetone. Additionally, electrons were donated to the ZnO domain by removing the adsorbed oxygen species close to the interface. In response to this charge restructuring, the charge depletion area moved farther into the CuO domains. A considerable increase in resistance was observed across the CuO surface compared with the nanocubes without ZnO cores [134,135,136].

### 3.5. Intrinsic Physical Characteristics

Several physical characteristics affect the application of SMOx materials in numerous fields, including morphology, particle size, crystal face, and porosity. A SMOx-based sensor, for instance, has nanoscale pores that can act as barriers to small grain sizes and improve the electronic transmission of sensitive receivers, thus influencing sensitivity to gases [36,137]. In order to diffuse gas molecules into the sensitive receptor, SMOx surface pores should be large. It has excellent sensitivity characteristics because it has large pores on the surface and small pores on the bulk, and it has good grain boundary contact [138,139]. 

## 4. Gas-Sensing Applications

**Figure 4:**
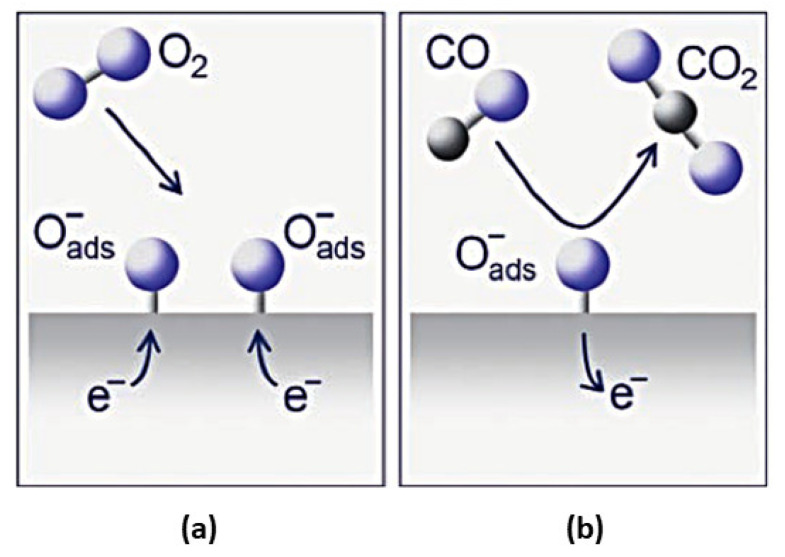
Schematic illustration of the chemical reactions at the surface of an n-type gas sensor. (**a**) Chemisorption of oxygen (O_2_) traps electrons from the conduction band and forms the charged species atomic O^−^ and molecular O_2_^−^. (**b**) Reducing gases (e.g., CO) react with the surface-bound oxygen and release electrons back into the crystal leading to changes in the electrical conductivity that are related to the CO concentration [159].

A gas sensor fabricated with a gas-sensing element can detect analytic gas species by converting surface interactions into electrical signals [139]. In the last few decades, metal oxide (MOx) gas sensors with a simple and cost-effective fabrication process, high sensing response, and short recovery time captivated the attention of researchers because of their excellent surface morphology, high structural stability, adaptable electrical properties, and grain size, where metal oxide is used as the chemosensory material [5,140,141,142,143]. In 1960, Seiyama et al. introduced the gas-sensing properties of ZnO thin films, which have been used and studied extensively since then [144,145]. The advancement of gas sensing technology has allowed modern gas sensors to operate at low power [146,147,148,149,150,151,152]. As a result of chemical reactions between gas molecules and semiconductor metal oxide surfaces, semiconductor-metal-oxide-based gas sensors have great potential (Figure 4) [144,153,154].

SMOx-based chemoresistors are portable devices that use a battery to operate at high temperatures. In factories, plants, and industries, these devices are used for detecting hazardous gases, for example, oxygen control in the exhaust emissions of gasoline, diesel, and gas engines, and humidity and air quality control in automobiles [155,156]. SMOx-based gas sensors are more sensitive when dopants are used [157], and the electrical properties of SMOx depend on the interactions between the sensor surface and gas molecules adsorbing on it [158].

### 4.1. Mechanism of SMOx-Based Gas Sensors

The sensing mechanism of a SMOx is complicated by different key factors that affect the sensing attributes, including the adsorption ability, electrophysical property, catalytic and chemical activity, thermodynamic stability, and surface adsorption or desorption properties [30,160,161,162,163,164]. Two processes are involved in the sensing mechanism of SMOx-based gas sensors: reception and transduction [19,156]. In the reception process, the sensor converts the chemical reaction into energy, which is then converted into analytical signals in the transduction process [165]. Through the gas–solid interface, the target gas is detected on the SMOx surface through a change in electrical properties. In the transduction process, chemical changes are induced in the surface and transformed into electrical signals, such as resistance changes in the sensor [166]. As a result of reversible redox reactions between reactive gases and the SMOx surface, a SMOx’s electrical properties change. Finally, the electrical signals were measured and displayed using suitable circuits, such as a microprocessor unit [12,167].

The detection of gas molecules is primarily carried out by monitoring variations in the device current (IDS) or threshold voltage (VTH) caused by the adsorption of nearby molecules. In the case of Ohmic connections, such changes can occur by modulating the conductivity of FET channels, and in the case of non-Ohmic links, by modifying the Schottky barrier height. Due to the conductivity of the FET channel, the free-carrier density in 1D/2D channels is decreased or increased during this process. If the hole (electron) carrier density alters due to gas adsorption, the phenomenon is called hole (electron) doping. An adsorption-induced doping process results in a positive (electron-acceptor) or negative (electron-donor) shift in the VTH, which, in turn, causes an alteration in the IDS for a VDS value. The adsorption of molecules from the surrounding environment can increase or decrease the charge carrier surface scattering and trapping. A change in the majority carrier mobility (p for holes and n for electrons) affects the channel conductivity for both p- and n-type FETs, modulating the IDS. A Schottky contact also modifies the metal workfunction, affecting the height of the energy barrier at the semiconductor interface [6,168].

To evaluate the response and recovery times of a FET sensor as a result of gas molecule adsorption/desorption processes, the real-time measurement of the IDS for VDS and VGS values was used. Trans-characteristic analysis revealed different sensing mechanisms for a 1D and 2D FET. FETs have the advantage of simultaneously monitoring many electrical parameters, such as the IDS, VTH, SVTH, SW, and Ion/Ioff upon gas molecule adsorption, over two-terminal electrical devices (such as resistors, capacitors, and diodes). This can be used to retrieve data on the specific sensing mechanisms when target analytes interact with the FET, such as changes in the concentration of electrons/holes, the energy barrier at the semiconductor/metal interface, or the mobility of the majority of charge carriers. As a result, scientists can tweak the gadget’s architecture and materials to enhance its performance. Aside from this, the sensitivity of the sensor can also be electrically tuned for the detection of low (by lowering the amount of charge in the channel and, consequently, the background current) and high concentrations of the target gas by varying the VGS value and the charge carrier concentration in the FET channel, as opposed to two-terminal electrical components, which prevent it from being effective [6].

**Reception:** On the SMOx surface, the reception process involves the reactions (i) ionosorption of oxygen to form reactive oxygen species and (ii) the reaction of these reactive oxygen species with reducing gases. When SMOx-based sensors are heated without oxygen, free electrons easily flow through their boundaries. A SMOx (e.g., SnO_2_), on the other hand, forms a potential barrier when oxygen is adsorbed onto its surface due to its presence in the atmosphere. Through this interaction, atmospheric oxygen traps electrons from the bulk material, thereby depleting a region of electrons. As a result, an increased potential barrier is formed at the surface. The flow of electrons is impeded by this phenomenon, which increases the resistance. The surface of the SMOx sensor absorbs gas molecules when exposed to reducing gases, lowering the potential barrier and allowing electrons to flow more freely. As a result, the electrical resistance also decreases. SMOx-based sensors were demonstrated to be variable resistors. 

**Figure 5:**
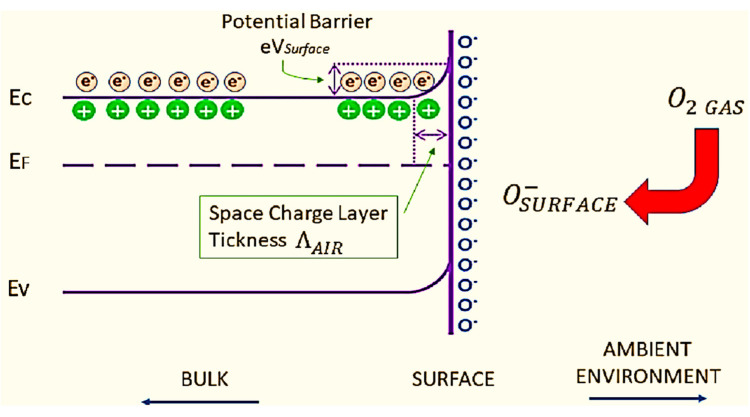
Schematic view of band−bending after the ionosorption of oxygen (chemisorption), where EC, EV, and EF denote the energies of the conduction band, valence band, and Fermi level, respectively. “e^−^“ represents conducting electrons and “+” represents donor sites [13].

Due to its wide band gap, SMOx has many electrophysical properties, including insulating behavior and semiconductor properties [169,170,171,172]. A change in conductivity is caused by electrons trapped in adsorbed molecules. Figure 5 [13] shows that electrons are extracted from the conduction band (Ec) when oxygen molecules (O_2_) adsorb on the SMOx surface [173,174,175] using ionosorption [173,174]. A space charge layer is formed when this phenomenon causes an upward band bend, resulting in an electron-depleted region. By reacting with CO, oxygen species decrease the amount of adsorbed oxygen at the surface, which reverses the band-bending process (Reaction 2). In addition, SMOx gas sensors display increased conductivity with high temperatures (300–450 °C) [25]. In n-type semiconducting metal oxides, where the depletion region is smaller than the grain size, this mechanism is crucial and well-suited for sensing gases.

(1) $$\frac{1}{2}O_{2}+V_{0}^{++}+2e^{-}\;⇋{\;O}_{0}$$

where $V_{0}^{++}$—oxygen vacancy.

(2) $$O_{0}+\mathrm{CO}\to {\mathrm{CO}}_{2}+V_{0}^{++}+2e^{-}$$

**Figure 6:**
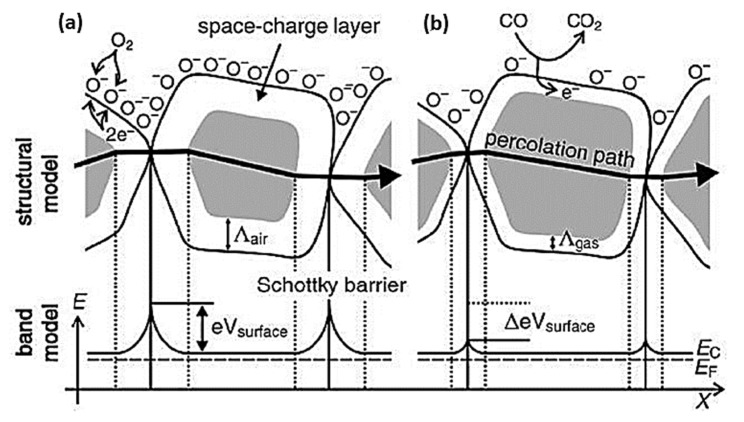
The structural and band models in the (**a**) presence of CO and (**b**) absence of CO [13,176].

SMOx gas sensors become oxidized when exposed to a reducing gas, such as CO, releasing electrons into the bulk material, resulting in a decrease in the number of O^−^ ions on the surfaces (Figure 6). As a result, the thickness of the space charge layer is reduced. By doing so, Schottky barriers between grains or particles become smaller, allowing electrons to easily pass through sensing layers.

Depending on the atmospheric composition, the surface reactions vary, which, in turn, changes the concentration of trapped charges on the surface and the associated space charge layer [177,178].

**Figure 7:**
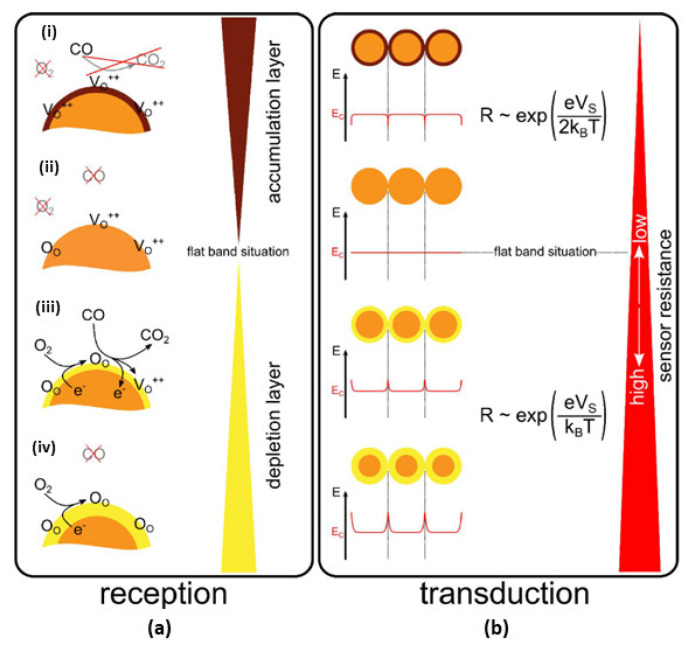
(**a**) Sensing mechanism of pristine n-type SMOx materials under four conditions in the reception process: (i) formation of an accumulation layer (brown color) in the presence of reduced gas with the absence of oxygen, (ii) flat band formation in the absence of surface states due to adsorbed species, (iii) formation of a depletion layer (yellow color) in the presence of oxygen and reducing gases, and (iv) formation of a depletion layer without reducing gas in the presence of oxygen. (**b**) Charge transport in the sensing layer depicting tentative resistance in the transduction process [20].

**Transduction**: It is necessary to convert surface charges into measurable electrical signals in order to obtain analytical signals. SMOx-based gas sensors measure surface reactions by varying the sensing layer’s resistance. The surface charge is affected by the structural and morphological properties of the sensing layer [80,178], the electrical and chemical properties of SMOx, the size and shape of the SMOx material [179,180,181], and the electrode’s geometry [12]. According to Figure 7, spherical SMOx grains with a Debye length smaller than the grain radius were not affected by changes in the surface, resulting in an unaffected bulk region. A continuous Schottky barrier is created between different grain contacts at the surface as a result of the band bending. Due to oxygen biosorption, an electron-depleted surface layer formed in n-type SMOx, whereas a hole-depleted surface layer was observed in p-type SMOx [166,178,182].

**Figure 8:**
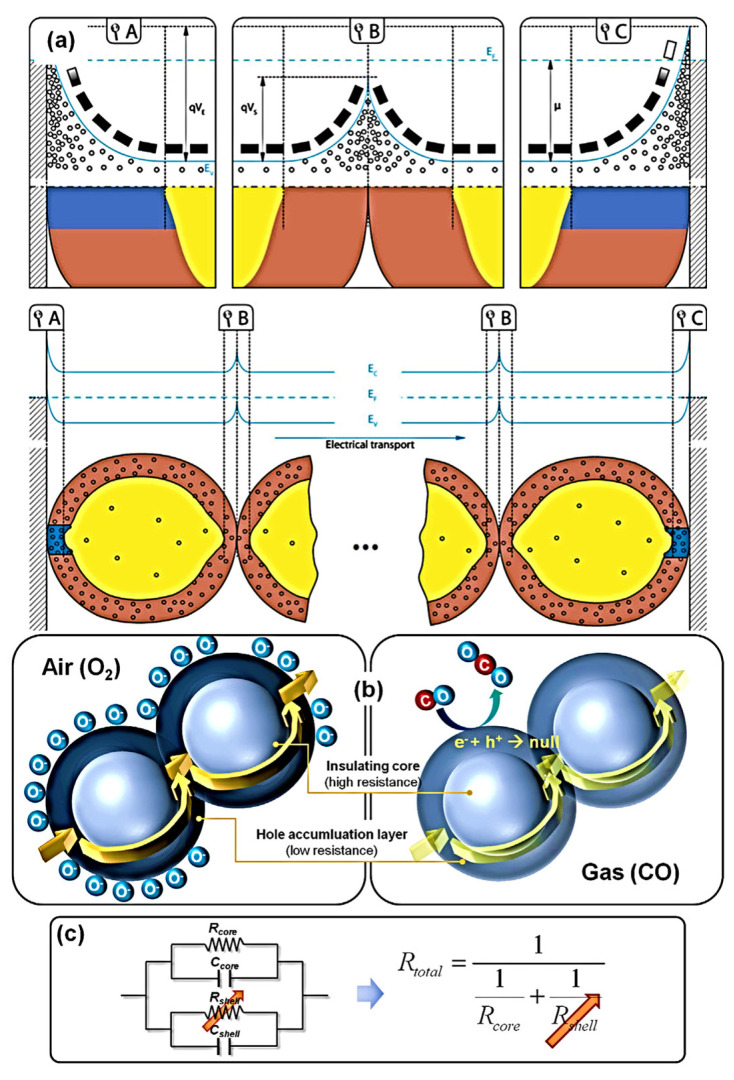
(**a**) Simplified representation of the essential sensing layer components for p-type oxide semiconductor gas sensors (low), where A and C are metal–semiconductor contacts and B is a grain-grain contact of a semiconductor. The energy band diagram described by Barsan and co-workers [166]: (**b**,**c**) p-type oxide semiconductors with a simplified gas-sensing mechanism and equivalent circuit [182].

Conduction in p-type oxide semiconductors might be explained by a conflict between parallel routes spanning a broad resistive core (Rcore) and a constrained, p-semiconducting shell (Rshell). Barsan and co-workers provided a detailed explanation of the precise conduction model and energy band diagram of p-type oxide semiconductor-based gas sensors (Figure 8a) [166,182]. As shown in Figure 8a, the B region represents the electrode–semiconductor connections, while the A and C regions show the semiconductor grain-to-grain interactions. Oxygen anions on the surface of the SMOx react to form an oxidation process that injects electrons into the material, reducing the concentration of holes in the shell layer while increasing the sensor resistance (Figure 8b,c).

P-type oxide semiconductors are known to lose resistance when exposed to oxidizing gases like NO_2_ and O_3_. As a result of the ionosorption of oxidizing gas, the concentration of holes in the shell layer increases. The chemo-resistive variation of p-type oxide semiconductors to oxidizing gases also appears to be not high when considering the gas-sensing mechanism. Based on literature data, NiO and CuO sensors respond moderately to 10–100 ppm NO_2_ (Ra/Rg = 1.0–7.5) [183,184,185].

### 4.2. Sensitization Mechanism

**Figure 9:**
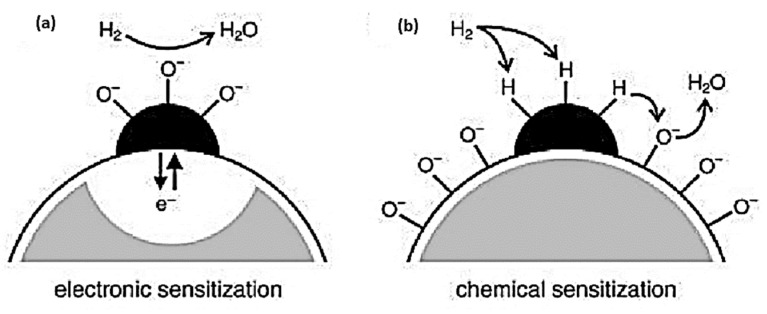
Sensitization mechanisms of SMOx by metal or metal oxide additives. (**a**) Electronic sensitization via changes in the Fermi level and (**b**) chemical sensitization via spillover [13].

The sensitization mechanism enhances the sensitivity of gas sensors by adding additional material or by modifying the existing material. The variation in the reception and transduction mechanisms increases the electronic and chemical interactions between the target gas and sensing material, resulting in a high sensitivity of the sensor [186,187,188]. As a result of dividing sensitization mechanisms into two main categories, namely, electronic sensitization and chemical sensitization, N. Yamazoe developed the concept of clear separation of electronic and chemical sensitization. By adding metal or metal oxide additives, both sensitizations could be achieved. During electronic sensitization, the additive accepts electrons from the analyte and changes its redox state or chemical potential (Figure 9a). Chemical sensitization, on the other hand, involves activation of the analyte, spillover, and a change in the surface oxygen concentration (Figure 9b). The concentration of charged species at the surface and space charge layers remains unchanged without an analyte. As a result of the high surface concentration of active oxygen, a different phenomenon occurs during oxygen spillover. The high concentration of charged species at the surface of SMOx causes the band bending to increase when chemical activation occurs.

**Figure 10:**
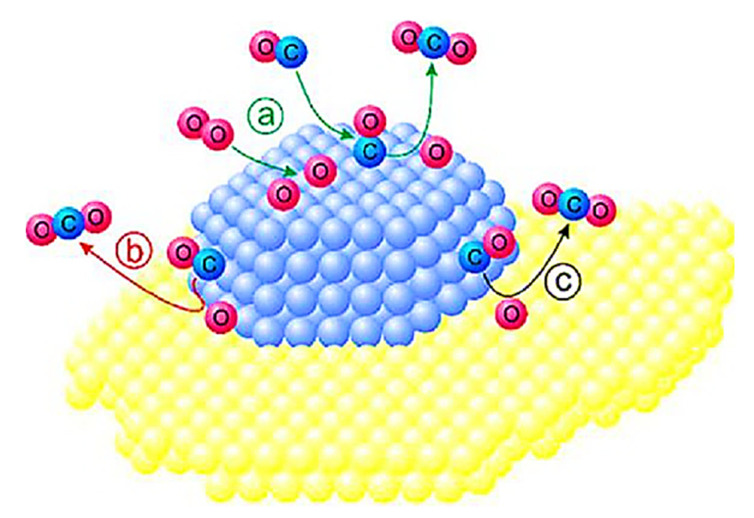
Schematic view of catalytic activation based on oxidation mechanism. (**a**) Direct reaction on the additive surface; (**b**) adsorption of oxygen on SMOx additive; (**c**) spillover of reactive species on the SMOx surface. O = oxygen (red colour), C = carbon (blue colour) on metal nanoparticle surface [189].

**Figure 11:**
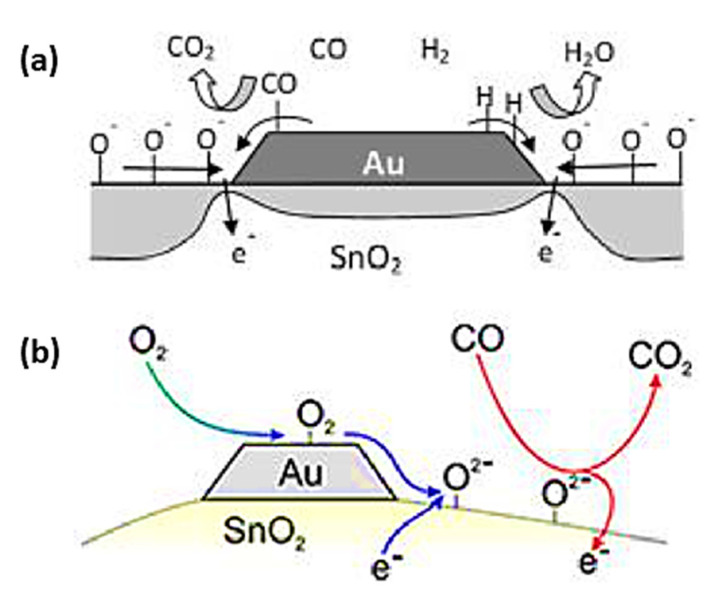
Schematic view of spillover mechanism on a Au− loaded SnO_2_ gas sensor where (**a**) inverse oxygen spillover and (**b**) oxygen spillover take place [191,197].

Spillover effects promote the separation of molecular oxygen into an active surface. Spillover activation significantly impacted the sensor’s gas-sensing properties. As the analyte gas adsorbs on the additive phase, the activated species are transferred to the SMOx surface, where the analyte gas reacts with active oxygen species (Figure 10). The oxidation catalysis process is affected by single additive sites, i.e. dopants [189] and by separate additive phases [190,191,192]. As a result of spillover effects, molecular oxygen separates into a more active surface. Chemical sensitization involves two important aspects: (i) the change in the surface charge of SMOx caused by chemical activation and (ii) the ambivalent relationship between catalytic activity and gas sensing. As an example, CO is activated by adsorption and transfer to SMOx surfaces, while other analytes (e.g., H_2_) are activated by additive reactions. Analyte gas spillover activation enhances the reactivity of SMOx and accelerates oxygen vacancy formation. Despite the inverse spillover mechanism described by Korotcenkov et al. for the detection of reducing gases with Au-loaded SnO_2_ (Figure 11a) [191], spillover mechanisms are generally used to improve the gas-sensing properties of metal-loaded SMOx (Figure 11b) [186,193,194,195,196].

The increase in initial band bending directly affects the resistance change due to changes in the surface charge of SMOx, which shows a significant impact on the electrical and chemical properties of the SMOx surface, and consequently on transduction and reception processes. The initial band bending is described by a non-linear relationship between the surface charge (*Q_S_*) and the surface potential (V_S_). The Schottky approximation, described by S. R. Morrison, provides a simplified explanation of this relationship [198] as follows:

(3) $$V_{s}=\frac{e}{2\varepsilon \varepsilon_{0}n_{b}}\times Q_{S}^{2}$$

where

*e*—elementary charge;

*ε*—permittivity of SMOx;

*ε*_0_—permittivity of vacuum;

*n_b_*—concentration of charge carriers in the bulk.

The sensor signal (S) for the reducing gas is defined by the ratio of the resistance in the reference atmosphere (*R_ref_*) and the resistance in the presence of the analyte gas (*R_gas_*) [178]:

(4) $$S\;=\frac{R_{ref}}{R_{gas}}$$

The relationship between the differential surface potential $\Delta V_{s}$ and sensor signal S is as follows:

(5) $$S\;=\;\exp\;(\frac{e\Delta V_{s}}{k_{g}T})$$

The change in band bending at the surface, which is represented as the differential surface potential $\Delta V_{s}$ as a function of the change in surface charge $\Delta Q_{s}$ as follows:

(6) $$\Delta V_{S}=\frac{e}{2\varepsilon \varepsilon_{0}n_{b}}\times \Delta Q_{S}.\left(2\times Q_{S,0}-\Delta Q_{S}\right)$$

**Figure 12:**
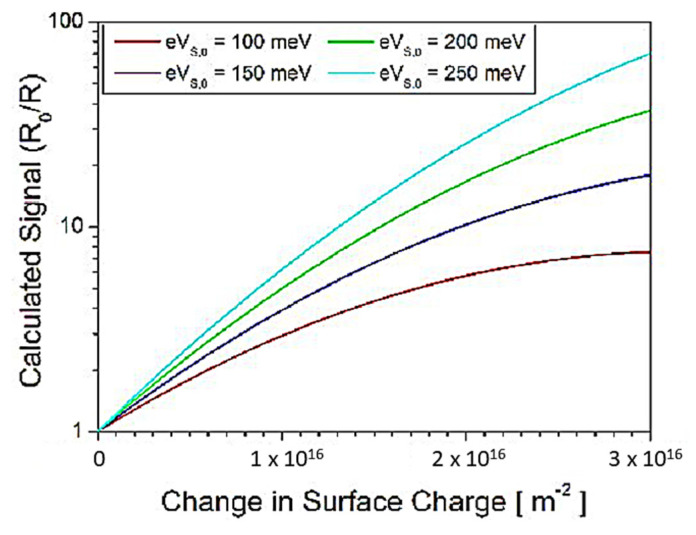
Sensor signal changes corresponding to changes in the surface charge for a SnO_2_—based sensor at 300 °C [199].

According to Equation (6), the change in the surface potential (Δ*V_S_*) is a function of the initial surface charge (*Q_S_*_,0_) and the change in the surface charge (Δ*Q_S_*) as a consequence of reactions taking place on the surface. Combining Equations (5) and (6), the impact of different *Q_S_*_,0_ values on the sensor signal can be calculated. From Figure 10, it is evident that the change in the surface charge results in a change in the sensor signal. The slopes of the curves indicate that a higher initial band bending leads to an increased sensor signal and hence sensitivity. The increase in slopes, referred to as n-values, is experimentally observed in SnO_2_ materials due to the self-doping effect (Figure 12) [199]. The presence of two SMOx materials shows a major effect on the electronic coupling between them, as the second material provides additional reaction sites on the additive phase [187,188].

### 4.3. Factors Affecting the Sensitivity

#### 4.3.1. Chemical Composition

The chemical composition of SMOx plays a significant role in improving the adsorption ability, catalytic activity, sensitivity, and thermodynamic stability [200]. Recently, composite materials, like SnO_2_-ZnO [201,202], Fe_2_O_3_-ZnO [203], and ZnO-CuO [204], were investigated for their improved performance. Furthermore, researchers are working on various ternary, quaternary, and complex metal oxides for different applications [205,206]. The combination of metal oxides and other components, for example, organic and carbon nanotubes, also showed promising results. Therefore, the chemical composition significantly influences the gas-sensing properties of composite metal oxides.

ZnO-SnO_2_-composite-based sensors exhibit higher sensitivity compared with sensors made solely from tin dioxide or zinc oxide [203]. As described by De Lacy Costello and colleagues, the combined effect of two components in a synergistic effect enhances the sensitivity of sensors [203].

#### 4.3.2. Surface Modification

**Figure 13:**
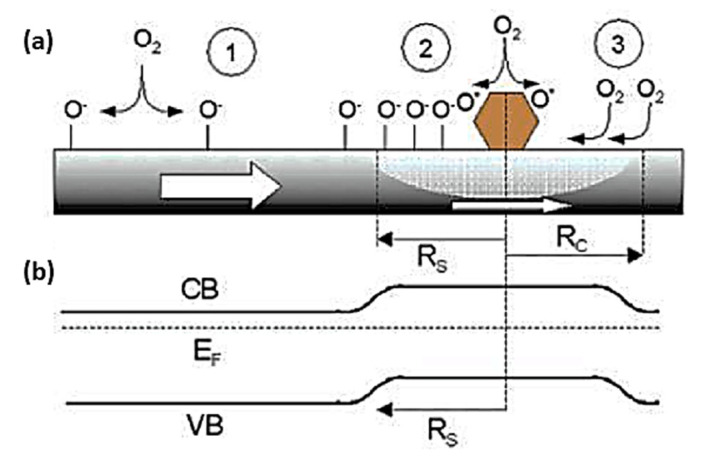
(**a**) Schematic view of a SnO_2_ nanowire surface in the presence of O_2_ and (1) ionosorption of oxygen at defect sites of the pristine surface; (2) molecular oxygen dissociation on Pd nanoparticles followed by spillover of the atomic species onto the oxide surface; (3) capture by a Pd nanoparticle of weakly adsorbed molecular oxygen. (**b**) band diagram of pristine SnO_2_ in the vicinity of a Pd nanoparticle. The radius of the depletion region is determined by the radius of the spillover zone [216].

Controlling the catalytic activity of gas sensors is another way to enhance their performance. Several widely used SMOxs, such as TiO_2_, ZnO, SnO_2_, Cu_2_O, Ga_2_O_3_, and Fe_2_O_3_, have low activities [160]. Without a catalyst, pure SnO_2_ exhibits poor sensitivity [207]. Noble metals, such as Pt, Au, Pd, and Ag, were highly effective oxidation catalysts and were also used to enhance reactions on metal oxide surfaces [208,209,210,211,212]. In order to detect Pd upon deposition, two mechanisms are involved: (i) electronic and (ii) chemical. The electronic mechanism involves the formation of depletion zones around the modified particles (Figure 13b) and modulation of the nano-Schottky barriers, which result from changes in the oxidation state of Pd during oxygen adsorption and desorption, boosting the sensing process. In Figure 13a, oxygen is shown as ionosorbing on the surface of Pd. Pd, which catalyzes molecular oxygen dissociation, is a better oxygen dissociation catalyst than tin oxide. As a result, the atomic products diffuse to the surface of the SMOx (Figure 13a) [213]. Oxygen molecules reside on the oxide and diffuse to the catalyst particle. A back-spillover effect (Figure 13a) and effective “capture radius” (Rc) are observed around Pd particles (Figure 13b). An oxygen delivery system is developed when oxygen layers cover whole metal oxide surfaces [214]. This process enhances the oxygen ionosorption on MOx and the detection sensitivity [215].

#### 4.3.3. Microstructure

**Figure 14:**
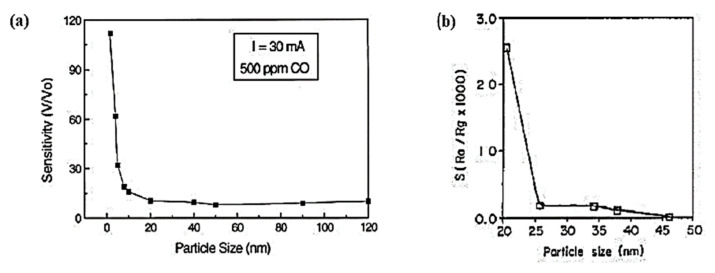
(**a**) Effect of particle size on CO gas sensitivity [225]; (**b**) effect of particle size on H_2_ gas sensitivity [217].

A significant effect on sensitivity was also observed when metal oxides were synthesized with an optimal morphology and crystallographic structure. SMOx sensors are made more sensitive by using small-grain materials in this method. According to Lu et al. the sensitivity of a SnO_2_-based sensor to 500 ppm CO increased dramatically for particle sizes smaller than 10 nm (Figure 14a). Similarly, 20 nm particles demonstrated ten times more sensitivity compared with 25~40 nm particles (Figure 14b) [217]. In the case of small grains with narrow necks, this size is less than twice the thickness of the surface charge layer [218]. In metal oxide gas sensors, the size of the grains affects the mobility of free charge carriers, and therefore, the number of collisions that occur between them. Additional scattering centers are formed by adsorbed species, which influence carrier mobility as well [178]. As a result of this method, metal oxide gas sensors are significantly more sensitive [178,219,220,221,222]. It is worth noting that using a small crystal size did not always enhance the gas sensor’s response [223]. Sensors fabricated with SnO_2_ nanocrystals (50 nm) synthesized via gel combustion showed a faster response than those fabricated with SnO_2_ nanocrystals (12–13 nm) synthesized via the hydrothermal method. In contrast with hydrothermally synthesized SnO_2_ nanocrystals, which consist of small grains but tend to aggregate into large entities, gel-combustion-synthesized SnO_2_ nanocrystals were more porous. As a result, tuning the grain size of metal-oxide-based gas sensors could enhance their performance. In addition to modulating structural stability, grain size affected the surface changes, as well as the catalytic activity [224].

#### 4.3.4. Humidity and Temperature

**Figure 15:**
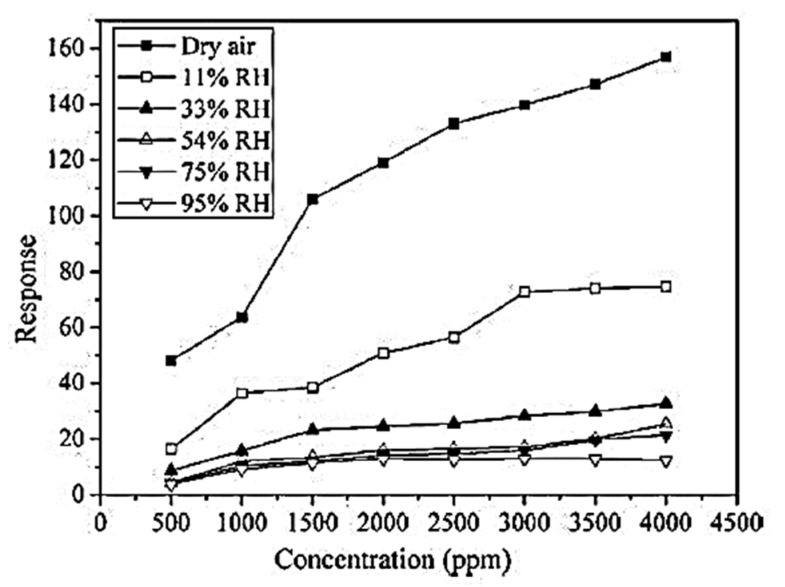
Response of Sm_2_O_3_ doped SnO_2_ sensor for different concentrations of C_2_H_2_ at different relative humidity (RH) conditions [228].

A significant role was played by humidity in modulating the activity of metal-oxide-based gas sensors. A variety of humidity sensors based on metal oxides have been developed. In contrast, the mechanisms of sensing water vapor and CO, NO_2_, and H_2_S gases differed. It was the ionic humidity sensor that was most commonly used with metal-oxide-based humidity sensors. H^+^ or H_3_O^+^ ions produced by the dissociation of adsorbed water on the surface are the conduction mechanism in metal-oxide-based humidity sensors. Recent works [226,227] studied and described the adsorption of water on metal oxide surfaces and the mechanism of sensing water vapor. As a result of water adsorption on metal oxide surfaces, humidity decreased the sensitivity of metal oxide sensors (Figure 15) [228,229].

**Figure 16:**
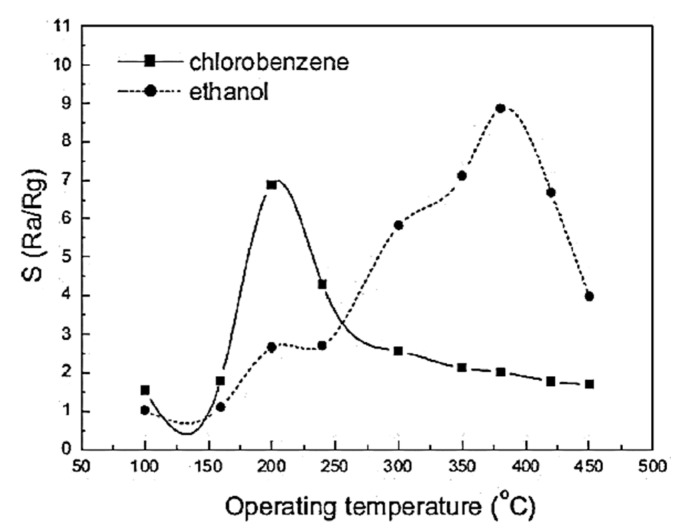
Response of a porous ZnO gas sensor as a function of operating temperatures [230].

Temperature is another important factor that affects the performance of metal oxide gas sensors. As shown in Figure 16, the sensor responses to different analytes show similar shapes as a function of temperature. The resultant shape depicted slow kinetics at low temperatures and increased desorption at high temperatures [216,230,231,232,233,234].

#### 4.3.5. Control Synthesis

**Figure 17:**
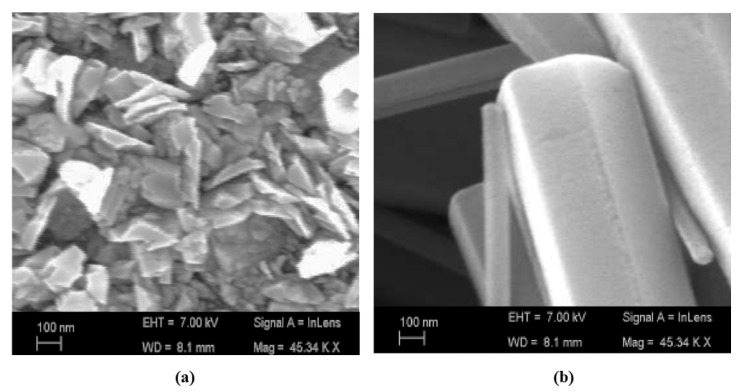
SEM images of (**a**) ZnO before a chemical treatment and a (**b**) hexagonal ZnO nanorod [236].

The ability to detect gas depends on its composition, shape, size, and distribution. Depending on the crystallographic orientation of the metal oxide semiconductor, sensitivity is improved. A higher sensitivity is observed for ZnO with the crystallographic orientation (002) [235]. For the crystallographic orientation, a high surface-to-volume ratio is also crucial to achieving greater sensitivity. Furthermore, a well-organized pore structure and particle size contribute to a high surface-to-volume ratio. ZnO was characterized using a scanning electron microscope (SEM) after it was synthesized (Figure 17a,b). Surface morphology can be modified via chemical processing and variable annealing temperatures. As a result, hexagonal-shaped ZnO was developed (Figure 17b). A hexagonal shape with a high surface-to-volume ratio produces good sensitivity.

#### 4.3.6. Doping with a Noble Material

To assess conductivity, the effectiveness of catalytic reactions with target gas is measured on the surface of the sensing material. It is also important to improve catalytic activity in order to improve sensor performance. Undoped metal oxides exhibit significantly lower activity than doped ones [237]. Sputtering, thermal evaporation, or sol-gel doping could be used to dope semiconductors. A surface modification sometimes requires the combination of noble materials and MOxs (metal oxides). In addition to Pd, Ag, Au, and Pt nanoparticles, second-phase nanoparticles with enhanced sensitivity were applied to host metal oxides [238]. As a result of the catalytic behaviour of noble metal nanoparticles, the chemical dissociation and reactions are increased [239]. The activity of Pd-functionalized SnO_2_ is improved [216]. ZnO nanostructures decorated with PdO also demonstrate improved sensitivity [230].

### 4.4. SMOx-Based Gas Detection for Environmental Monitoring

Here, we discuss a few SMOx-based gas sensors for monitoring the toxicity of gases in the environment, industries, hospitals, health issues, etc.

#### 4.4.1. Nitrogen Oxide Gas Detection

WO_3_ sensors based on SMOx have been extensively used in gas sensors [240,241,242,243,244,245]. Most of the modified WO_3_-based metal composites were used for NOx sensing [246,247,248,249]. Modified thermal evaporation techniques were used to obtain nanostructured WO_3_ films with high surface roughness [240]. This technique produces high-response sensors with high selectivity and short response times, particularly at low temperatures (minimum 100 °C). Due to a high variation in electrical resistance, sensors at this temperature exhibited high sensitivity to NO_2_ with a low detection limit (around 100 ppb). In contrast, low responses were observed when NH_3_ (10 ppm) and CO (400 ppm) were present at high concentrations [240]. WO_3_ sensing elements for high-temperature potentiometric NOx sensing were synthesized by Yang et al. using various synthetic methods. Mixed oxides based on WO_3_ are also used for sensing. SMOx materials such as WO_3_-Ti [246,247,248], WO_3_-Pd, Pt, Au [249,250,251,252], WO_3_-In_2_O_3_ [253], and WO_3_-Bi_2_O_3_ [254] are used to fabricate selective and sensitive NOx gas sensors. Furthermore, TeO_2_-based thin films were synthesized for NO_2_ gas sensing [255,256]. As the NO_2_ gas concentration increases, the response time decreases. A response time of about 6 min for 1 ppm of NO_2_ gas and about 1.2 min for 120 ppm was reported. The recovery time was found to be longer than 8 min for each gas concentration [255].

The direct printing of numerous SMOxs, including ZnO, In_2_O_3_, SnO_2_, and WO_3_, was proposed by Kim and co-workers for the creation of an aligned network of NWs as the channel of a FET for NO_2_ detection [257]. The diameters of the manufactured NWs, which had aggregates of grains between 5 and 15 nm, ranged from 100 to 2400 nm depending on the concentration of metallic precursor in the printing solution. It was investigated whether a ZnO FET could detect NO_2_ at concentrations ranging from 1 to 5 ppm using an aligned network of NWs directly printed on Pt interdigitated source/drain electrodes. The sensing capabilities of a ZnO FET at concentrations ranging from 1 to 5 ppm were tested using an aligned network of NWs printed directly on Pt sensor electrodes. A rise (fall) in NW resistance was observed after the injection of 5 ppm of NO_2_ (50 ppm of ethanol) due to the target gas acting as an electron acceptor (donor) on n-type NWs. The sensor calibration curve (resistance variation vs. NO_2_ concentration) was linear in the tested range. An extrapolated LoD of 53.5 ppt was calculated. In response to 5 ppm of NO_2_, the response and recovery periods were 67 and 11 s, respectively.

#### 4.4.2. SO_2_ Gas Detection

Sulfur dioxide (SO_2_) is a common air pollutant that can be detected using sensors. Polymeric sensing films [258,259,260], as well as liquid and solid electrolytes, are used to make these sensors. SO_2_ gas sensors based on SnO_2_ [261], SnO_2_ doped Pd [262], WO_3_ doped with various metals [211,263,264], and vanadium oxide modified with TiO_2_ [264] were also developed and tested. During the initial detection, Berger et al. examined the interaction mechanisms between SO_2_ and the SnO_2_ sensor interface [261]. As a catalytic additive, SnO_2_-based gas sensors contained 0.05, 0.1, 1, and 3 mol% Pd. In recent years, thick-film technology has been used to increase the operating temperatures to 600 °C [262]. The magnetron sputtering method was used to fabricate active layers of pure and Pt-doped WO_3_ on a micro hotplate substrate to detect sulfur compounds (SO_2_ and H_2_S). A compact tubular SO_2_ sensor based on a sodium superionic conductor and V_2_O_5_-doped TiO_2_ sensing electrode was described by Liang et al. [265].

#### 4.4.3. H_2_S Gas Detection

Due to their excellent performance in detecting hydrogen sulfide (H_2_S) gas, SMOx-based gas sensors caught the attention of researchers. H_2_S is a toxic gas with a threshold limit of 10 ppm, and concentrations over 250 ppm cause serious health problems, including death. Metal oxides are a model-sensitive material for H_2_S sensing [266,267] and other gases [36,268] because of their high sensitivity, quick response, and ease of integration. In contrast, SMOx-based sensors have low selectivity, are highly dependent on the relative humidity, and require high operating temperatures (above 100 °C). Metal oxides (MOx) are the most commonly used substance in chemo-resistive gas sensors, both in academic and industrial settings [36,268,269]. Hexagonal WO_3_ nanoparticles show the best selectivity to 10 ppm H_2_S at 200 °C, according to Szilagyi and co-workers [48]. If the operating temperature drops below a certain level, the response does not occur. Interlaced and condensed WO_3_ nanofibers can detect H_2_S gas at ppm levels, according to Niu and co-workers [31]. WO_3_ is also hindered by low selectivity or high operating temperatures when used to detect H_2_S gas [270].

These materials can be combined with CPs (conducting polymers) to improve their sensing capabilities while addressing some of their flaws, such as low selectivity and high temperature [271]. MOx/CP composites already demonstrated H_2_S detection from 0.05 to 1000 ppm. By lowering the temperature of the sensor, hybridization with MOx significantly improves the response quality and operating conditions of the H_2_S sensor. MOxs and CPs have a delicate synergetic effect due to the entanglement of their sensing mechanisms (from CPs, MOxs, and p-n heterojunctions), as well as numerous parameters that influence their effectiveness (e.g., synthesis, deposition, and morphology). Furthermore, environmental factors and long-term stability (>1 month) were too rarely studied, even though they are crucial for sensor applications [272]. Different SMOx-based H_2_S gas sensors were successfully modified using the following materials: WO_3_ and WO_3_-based materials [273,274,275,276,277], SnO_2_ [229,278,279,280,281], ZnO [282,283], copper oxide [280,284,285], platinum and palladium oxides [286,287], indium oxides [287,288], silver-based materials [289,290], titanium oxide [291], and cadmium oxide [292]. In dry and wet synthetic air with varying levels of humidity, WO_3_-based SMOx sensors responded strongly to H_2_S. The H_2_S sensitivity of some WO_3_ thin-film sensors is at the ppb level. A slight increase in conductance was also observed in the presence of humidity [276]. H_2_S can also be detected at room temperature by ZnO-based sensors down to 0.05 ppm [282].

#### 4.4.4. Amine Gas Detection

In many fields, like food processing, fertilizers, chemical technology, medical diagnosis, and environmental protection, it is extremely important to detect any trace amount of ammonia/amine. WO_3_ [293,294], copper-based materials [293,295], ZnO [296], SnO_2_ [297], iron oxide [298], and Cr_2_O_3_ [299] are well-known materials for functionalizing ammonia/amine detecting sensors. To produce ZnO films doped with various amounts of RuO_2_, thick films of ZnO were immersed in an aqueous solution of 0.01 M ruthenium chloride (RuO_2_) [296]. At operating temperatures between 100 and 350 °C, the doped ZnO sensor was exposed to 1000 ppm NH_3_. With increasing operating temperature, the response increased.

#### 4.4.5. Hydrogen Gas Detection

Among its potential uses are automobiles, electricity generation in fuel cells, medicine, space exploration, industrial chemical production, and food production. In the event hydrogen leaks into the air from storage tanks or valves, explosive mixtures can form, making hydrogen-monitoring devices necessary. Enhanced sensitivity and selectivity to H_2_ gas were demonstrated with a nanostructured SnO_2_ thin film doped with silver (Ag) and platinum (Pt). At 100 °C, nanocrystalline SnO_2_ shows a fast response time (around two seconds) and a quick recovery time (around ten seconds). In addition to their high sensitivity to H_2_ gas, porous SnO_2_ particles have a high surface area [300]. In today’s world, chemo-resistive gas sensors based on the IOT (Internet of things) are used as H_2_ sensors with low power consumption and a lower temperature [301]. A potential technique for SMOx-based gas sensors is self-heating, particularly for materials with NW shapes. Self-heating gas sensors can significantly reduce their power consumption from several W to nW levels. Power consumption reductions can significantly extend the life of sensors and save a lot of energy. This can be accomplished using single, arranged, and networked NWs. However, networked NWs are simpler to synthesize than single or ordered NWs, which makes them the most popular morphology for self-heating petrol sensors. The majority of self-heating MOx materials were reported to have NW morphology. In spite of the fact that self-heated gas sensors can display power consumption in the nW range, most of these sensors have very low response values [302].

MEMS gas sensors based on SMOxs have straightforward topologies, are easy to manufacture, and consume little energy. Using MEMS gas sensors results in both a reduction in gas sensor size and a reduction in power consumption for both gas sensors and electrical devices. In terms of power consumption, MEMS-based gas sensors trail self-heated gas sensors. As a result of gas sensing measurements still requiring high temperatures, MEMS gas sensors rely on an external heater. As a result, the problem remains difficult to solve. Despite the significant advances in the development of low-power-consumption-based gas sensors, there are still several challenges and issues to overcome in order to achieve high sensitivity, selectivity, long-term stability, and quick response/recovery times.

As a H_2_-sensitive medium, tungsten oxides with palladium or platinum catalysts display a color change from pale green to blue when hydrogen reduces them to tungsten bronze [295,303].

#### 4.4.6. Volatile Organic Compound Detection

Animals and plants are both affected by volatile organic compounds (VOCs) that cause chronic diseases, such as eye irritation, throat and lung problems, and cancer in humans. Many studies were carried out on modified SMOx sensing films for the detection of atmospheric VOCs, such as ethanol, acetone, hydrocarbon, and liquefied petroleum gas (LPG). Different materials are used to modify SMOx sensors, such as SnO_2_ and SnO_2_-based materials [228,304,305,306,307], WO_3_ and WO_3_-based materials [308,309,310], titanium oxides [310,311,312], zinc oxides [310,311,312], iron oxides [313,314], cobalt oxides [315], cerium oxide sensors [316], and copper-based materials [317]. By adding basic metal oxides, such as lanthanum oxide (La_2_O_3_), ethanol gas sensors can be made more sensitive. In the presence of La_2_O_3_ and WO_3_, ethanol gas undergoes dehydrogenation and dehydration over SnO_2_-based elements, respectively [318]. SnO_2_ doped with cadmium oxide (CdO) shows enhanced sensitivity to C_2_H_5_OH and H_2_ at 300 °C with a detection limit of several ppm in air [319]. Similar to MEMS/NEMS chemo-resistive gas sensors, the IoT also enhances C_2_H_5_OH sensitivity at low temperatures or room temperatures because of the high surface area and reduced power consumption. VOCs, such as acetylene, LPG, and aldehyde, can be detected by modified tin-oxide-based films. HCHO was reported to be stable and sensitive in a SnO_2_–NiO composite material [320]. According to Qi and co-workers, SnO_2_-based sensors modified with 6 wt% Sm_2_O_3_ were 16.8 times more responsive to C_2_H_2_ than SnO_2_ sensors. As an excellent C_2_H_2_ sensor, the Sm_2_O_3_-doped SnO_2_-based sensor showed a high sensitivity under various humid conditions [228]. It was also demonstrated that SnO_2_-based sensors can successfully detect LPG [210,306]. In_2_O_3_ NW FETs doped with Yb were proposed by Jun and co-workers [321]. A 4 mol% Yb-doped In_2_O_3_ NW FET exhibited *n* = 6.67 cm^2^ V^−1^ s^−1^ n-type behaviour. Based on the output characteristics of VTH = 3.27 V, SVTHSW = 0.5 V dec^−1^, and Ion/Ioff = 10^7^, linear and saturation zones were assessed. Undoped In_2_O_3_ NW FETs exhibited n-type behavior as well, with *n* = 10.82 cm2 V^−1^ s^1^, VTH = 10.26 V, SVTHSW = 2.5 V dec^−1^, and Ion/Ioff = 10^3^. Over the entire test range, the calibration curve of the 4 mol% Yb-doped In_2_O_3_ NW FET displayed linear performance. In_2_O_3_ NW FETs with undoped silicon were approximately three times less sensitive [6]. The following table illustrates the gas-sensing activity of SMOx-based nanomaterials, where t_res_ corresponds to the response time and t_rec_ corresponds to the recovery time. SMOx-based gas sensors are compared in Table 2 for comparison.

## 5. Chemical Sensing Applications

**Figure 18:**
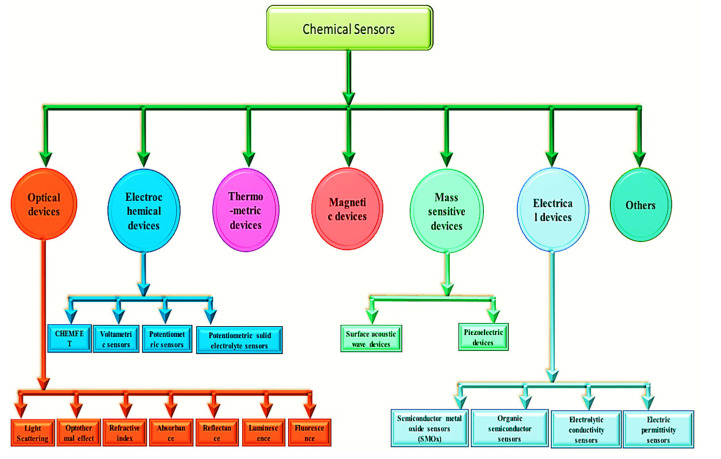
Schematic view of the classification of chemical sensors according to IUPAC [165].

Chemical sensors convert chemical reactions into electrical, optical, or mechanical signals by using chemical-responsive layers. Chemical sensors are more specific than physical sensors because of their chemical-responsive layers. In order for these sensors to respond, a chemical-selective layer must interact with the target chemical, changing the transducer properties and resulting in a signal. Conductometric chemical sensors based on SMOx were previously reported. As part of this development, MOx was discovered to react with the surrounding atmosphere, and the first commercial gas sensor was developed [144,354,355]. Their low cost, simple preparation, and simple operation make SMOx-based chemical sensors a promising technology. Chemical sensors were classified by IUPAC in 1991 (Figure 18). A chemical sensor is a device that converts chemical information, such as the concentration of a specific chemical component or a composite, into useful analytical information [165].

Chemical sensors have improved the detection and quantification of various chemical substances. Medical, agricultural, industrial, and military applications are all possible with these sensors.

**Figure 19:**
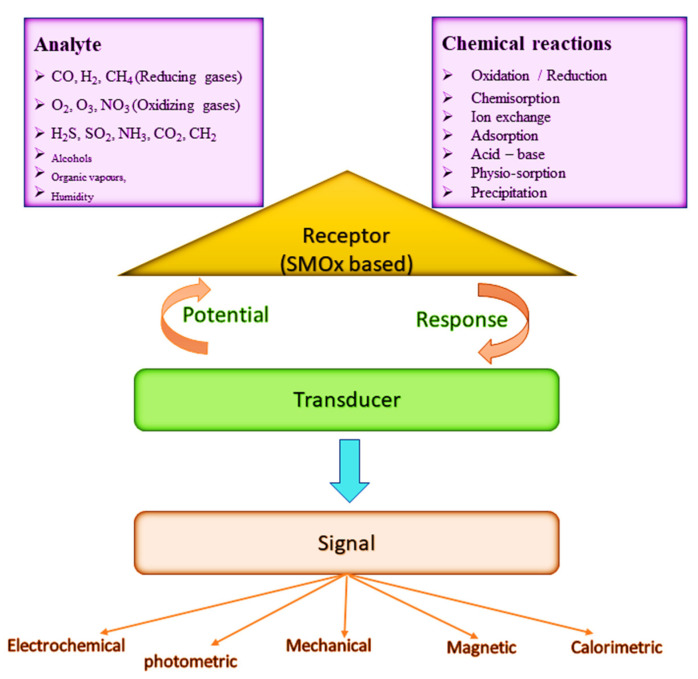
Schematic view of the principle of chemical sensors.

As illustrated in Figure 19, chemical sensors convert chemical information into quantitative or qualitative analytical signals through chemical interactions between the analyte gas or liquid and the sensor. Electric sensors produce signals through the exchange of electrons, which are electronic in nature. A chemical sensor consists of a physical transducer and a chemical-sensitive recognition layer. Stability, sensitivity, selectivity, response time, recovery, and saturation characterize them [155]. Due to their high sensitivity, compatibility with ambient conditions, and ease of fabrication, semiconductor metal oxides (SMOxs) are widely used as chemical sensing materials [163,356,357]. By exposing a metal oxide to elevated temperatures, the MOx reacts with surrounding gases, changing the surface potential and resistivity of the material.

Based on the MOx materials used for specific target species, SMOx-based chemical sensors are categorized.

### 5.1. SnO_2_-Based Chemical Sensors

SnO_2_ is a highly sensitive and fast-responding material that is widely used as a chemical sensor. Various morphologies are available, including nanowires, hollow spheres, nanocrystals, and others, each with its own unique sensing properties. Wang and co-workers reported that nanowires based on SnO_2_ were highly effective at detecting H_2_ at concentrations between 10 and 100 parts per million [358]. In addition, SnO_2_ nanowires, hollow nanospheres [359,360], nanocrystals [361], and nanocrystalline porous SnO_2_ [362] exhibit CO-, NO_2_-, and H_2_-sensing abilities. Hierarchical three-dimensional SnO_2_ nanospheres also exhibit excellent resistance to CO, methane, methanol, and ethanol [363]. Furthermore, SnO_2_ nano polyhedrons are highly sensitive to methanol, ethanol, and acetone, as well as highly selective to acetone, with a fast response and recovery time (only several seconds for target gas concentrations up to 200 parts per million) [364].

SnO_2_ is modified via doping and composite formation to enhance its sensing activity. In turn, this results in the development of highly sensitive and fast-responding SnO_2_-based composites, like polypyrrole-coated SnO_2_ hollow spheres, for the detection of ammonia [365]. Due to an increased concentration of oxygen vacancies on the surface of SnO_2_ nanowires at low temperatures, plasma-modified SnO_2_ nanowires [366] and Pt@SnO_2_ nanorods [367] displayed high sensitivity to ethanol gas. Adding Pt to SnO_2_ nanowires also enhances their chemical and electrical properties. For the detection of H_2_ gas (5 ppm) at 320 °C, Wang and co-workers synthesized hetero-junction p-NiO/n-SnO_2_ nanofiber-based sensors, which have excellent sensitivity and fast response recovery. Cu-doped SnO_2_ and the adsorption properties of H_2_S on the surface of SnO_2_ were investigated by Wei et al. [368]. Pd-doped SnO_2_-based CO gas sensors were recently described by Li et al. [369].

### 5.2. ZnO-Based Chemical Sensors

**Figure 20:**
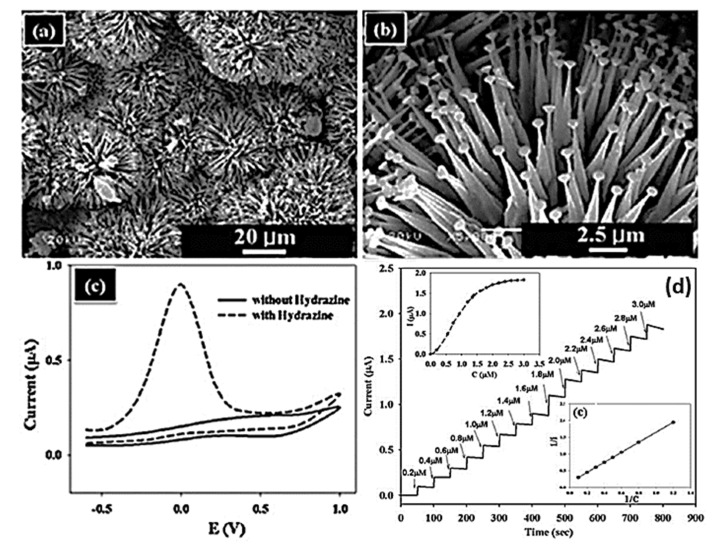
SEM images of ZnO material at (**a**) low magnification and (**b**) high magnification. (**c**) Cyclic voltammetry curve of a Nafion/ZnO/Au electrode in the absence of hydrazine (solid line) and presence of 1 mM N_2_H_4_ (dashed line) in 0.01 M phosphate − buffered saline (PBS) (pH = 7.4). The scan rate was 100 mV s^−1^. (**d**) Amperometric response of a Nafion/ZnO/Au electrode in the presence of hydrazine. The inset shows the 1/i versus 1/C plot [370].

Since they are easy to synthesize and have unique optical, electrical, and chemical properties, ZnO-based chemical sensors have received considerable attention. Using ZnO material, Hahn and co-workers fabricated hydrazine electrochemical sensors (Figure 20) [370]. Detection limits of 0.2 mM were achieved with this sensor’s high sensitivity (8.56 mA mM^−1^ cm^−2^) and low response time (less than 5 s). ZnO nanorods and high aspect ratio ZnO nanowires were also used by these researchers to fabricate hydrazine sensors.

**Figure 21:**
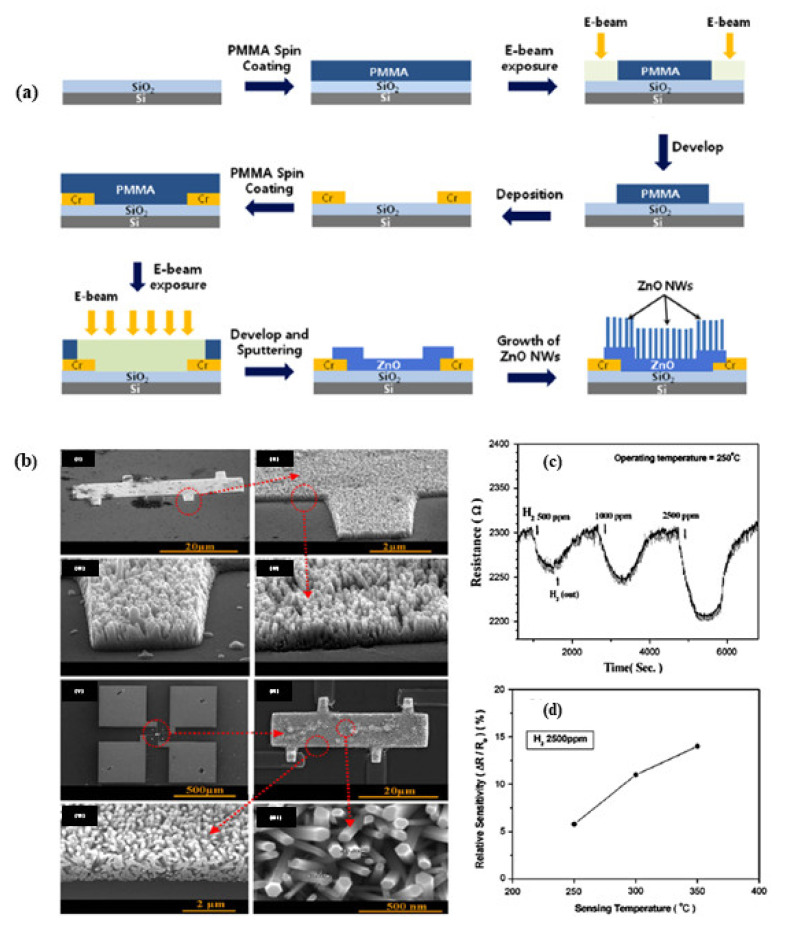
(**a**) Schematic view of the fabrication process of the electrode; (**b**) (i–iv) field emission scanning electron microscopy (FESEM) images of ZnO nanorod arrays (grown without electrode) and (v–viii) ZnO nanorod arrays grown directly on the probe; (**c**) dynamic responses of ZnO nanorod arrays to H_2_ pulses at 250 °C; (**d**) demonstration of the sensitivity at various temperatures [371].

The electrical characteristics of aligned ZnO nanorod arrays (NRAs) were also described by Hahn and co-workers (Figure 21) [371]. H_2_ was detected using ZnO NRAs. ZnO NRAs became more sensitive as the H_2_ concentration increased. Chemical sensors based on ZnO nanowires were also used to detect multiple gases at room temperature, including H_2_, NH_3_, i-butane, and CH_4_.

**Figure 22:**
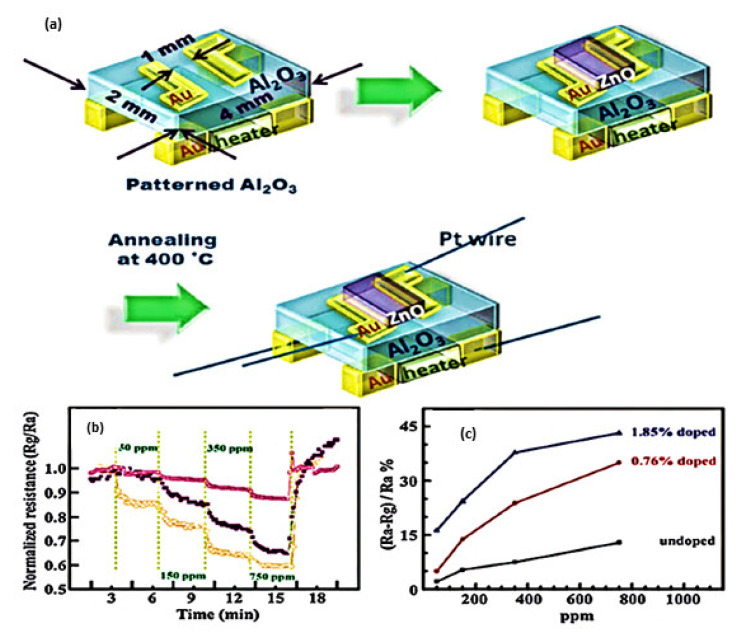
(**a**) Schematic view of the fabrication of a SMOx-based chemical sensor; (**b**) time-dependent resistance for continuous exposure of the sensor to CO at 350 °C; (**c**) sensitivity of undoped and 0.76% and 1.85% co-doped ZnO sensors exposed to different concentrations of CO [372].

As shown in Figure 22a [372], Li and colleagues synthesized co-doped ZnO nanorods on ITO substrates at low temperatures. As shown in Figure 22b, these nanorods responded rapidly to varying CO concentrations. In this study, co-doped ZnO sensors performed better than pristine undoped ZnO sensors. By attaching impurities to SMOx-based semiconductors, the sensing properties were greatly enhanced. Pd nanodots were incorporated into ZnO nanowires by Choi and Kim, which enhanced the CO sensitivity. A combination of electronic and chemical sensitization could be responsible for this enhanced sensitivity [373].

### 5.3. Other SMOx-Based Chemical Sensors

Chemical sensors rely heavily on selectivity. There is a slight deficiency of oxygen and incomplete crystallinity in gallium oxide (Ga_2_O_3_), which are preferred for high-temperature sensing applications, such as chemical, environmental, and explosive gas sensing [374]. Gallium oxide is chemically and thermally stable with low cross-sensitivity to humidity. The use of mesoporous single-crystal Ga_2_O_3_ nanoplates for CO detection was described by Yan and co-workers [375].

**Figure 23:**
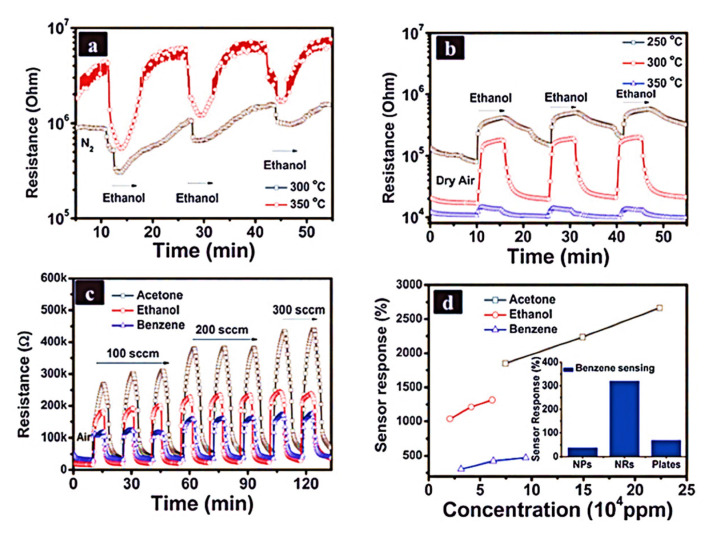
Gas-sensing performance of meso- and macroporous Co_3_O_4_ nanorod-based sensors. (**a**) ethanol sensing at different temperatures using N_2_ as the reference. (**b**) Ethanol sensing at different temperatures using dry air as the reference. (**c**) gas-sensing property of porous Co_3_O_4_ nanorods to acetone, ethanol, and benzene at 300 °C. The sensor resistance changes in response to different concentrations of acetone, ethanol, and benzene. (**d**) Response of nanoparticles (NPs), meso-/macroporous nanorods (NRs), and porous plates to different concentrations of acetone, ethanol, and benzene [376].

Using CoCl_2_ and urea precursors, crystalline mesoporous Co_3_O_4_ nanorod-based sensors were fabricated via facile hydrothermal methods. Benzene, acetone, and ethanol were detected using the sensor. In addition to its excellent stability, high sensitivity, rapid response, and recovery time, the SMOx-based sensor was particularly sensitive to acetone (Figure 23) [376]. The ethanol sensitivity of Fe_2_O_3_–TiO_2_ tube-like nanostructures was also improved [377]. In_2_O_3_-based chemical sensors were used to detect NH_3_, CO, H_2_S, NOx, ethanol, formaldehyde, and alcohol [378,379,380,381,382,383,384,385]. In Table 3, we present a comparative analysis of SMOx-based chemical sensors.

## 6. Biosensing Applications

**Figure 24:**
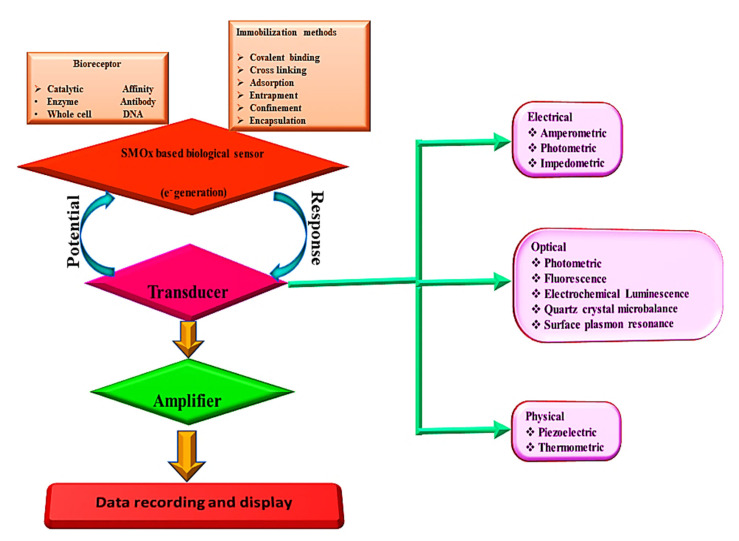
Schematic view of the principle of a biosensor.

Because of their exceptional electrical properties, high electron mobility, excellent chemical resistance in liquids, transparency, and ease of fabrication, semiconductor metal oxide (SMOx)-based thin-film transistors (TFTs) are widely used in liquid crystal displays (LCDs), biosensors, and photosensors [403,404,405,406,407,408]. SMOx-based biosensors are highly effective at recording and communicating biomolecule progression statistics [409]. Its versatile morphology [410], chemical stability [411], physicochemical interfacial properties [412,413], light excitation, and ability to form composite structures [412] make SMOx materials potential candidates for biosensors. Electrochemically sensitive materials, such as TiO_2_ [414], WO_3_ [415], SnO_2_ [416], and ZnO [417], are suitable for enzyme-based biosensors. In addition, these SMOx materials require a cost-effective synthesis procedure, including co-precipitation [416], chemical precipitation [418] thermal oxidation [419], chemical etching [420], polyol [421], hydrothermal [422], sol-gel [423], and sonochemistry [424], which allows for the formation of different architectural morphologies, including porous quasi-nanospheres [425], hollow nanospheres [426], nanorods [427], nanosheets [428], and flower-shaped particles. Additionally, SMOx materials can be combined with other materials to form heterostructures [429] hybrid structures [430], and composite structures [431]. Their advanced electrochemical properties make them ideal for specific biosensor applications. Biosensors require a sensing layer that reacts with a biomolecule, and this reaction is converted into optical, electrochemical, electrical, or other physical signals (Figure 24).

For clinical diagnostics and personalized care, biosensors must be sensitive. There are several reasons why nanostructured SMOx materials have excellent sensing capabilities: (1) their increased surface-area-to-volume ratio enhances sensitivity to small analytes, as their size becomes comparable to the SMOx materials [432,433] (2) direct electron transfer enables increased sensitivity and heightened detection limit [434]; and (3) nanostructured particles close to the Debye length, which increases their sensitivity [219]. SMOx surfaces are typically attached to biomolecules via physical adsorption, entrapment, crosslinking, covalent coupling, or encapsulation. In a biocompatible environment, such interactions form a nano–bio interface that is highly stable and preserves the biomolecules. Through the coupling of biomolecules with a bio-recognition layer, selectivity was achieved. A wearable biosensor continuously monitors physiological signals, collects sensor data, wirelessly transmits the data, and analyzes it in real time. Wearable biosensors have several advantages, including rapid continuous monitoring, detection of transient phenomena, ease of use, and accuracy. Following are a few promising nano-structured SMOx-based biosensors. Enzymes and other biomolecules are immobilized on the surfaces of these biosensors.

### 6.1. Enzyme-Immobilized Biosensors

**Figure 25:**
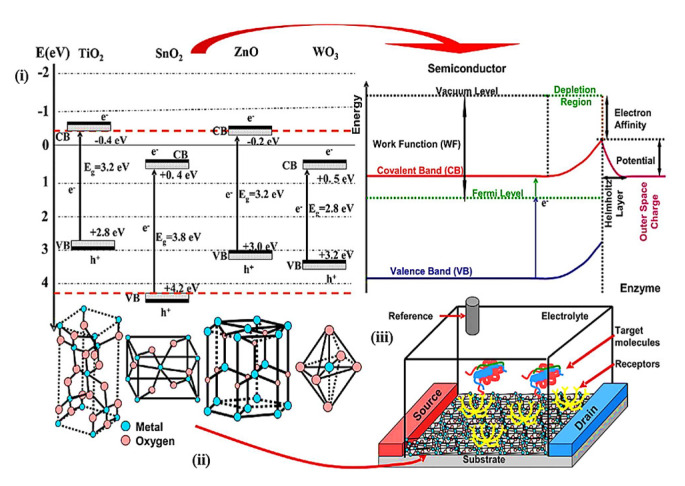
Mechanism of SMOx—based enzyme biosensors in three steps: (**i**) band energies; (**ii**) crystalline structure; (**iii**) configuration of an enzyme biosensor [32].

Figure 25 illustrates the energy band diagram and crystal structure of different types of SMOx biosensors [435,436]. SMOx properties can be modulated by combining them with other metal nanoparticles or ions. The morphologies of SMOx materials include rods, stars, flowers, cones, and porous or dense films.

#### 6.1.1. Glucose-Oxidase-Immobilized Biosensors

**Figure 26:**
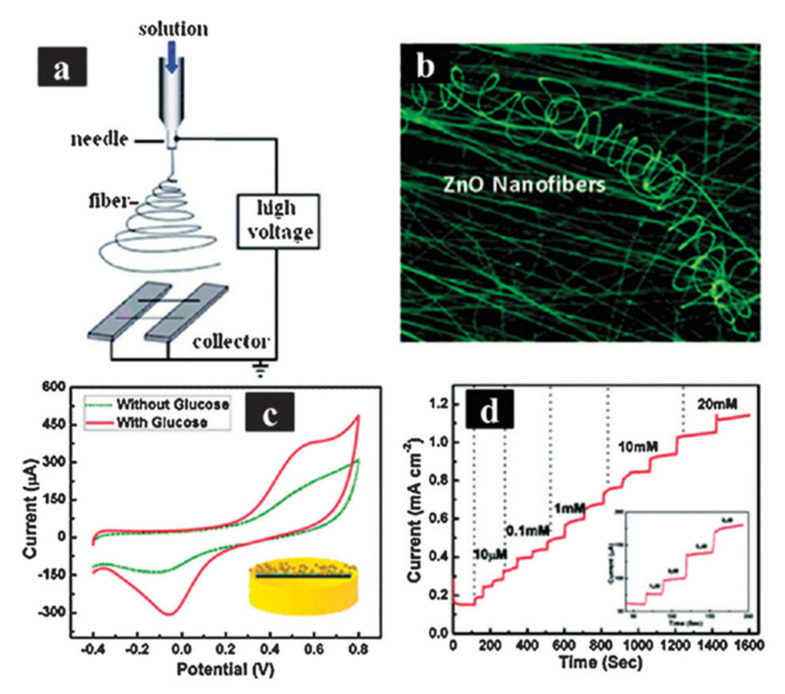
(**a**) Fabrication of zinc oxide nanoflowers; (**b**) SEM image of ZnO nanofibers; (**c**) cyclic voltammetry curves for a modified gold electrode without and with 100 mM glucose in PBS solution (pH = 7.0); (**d**) amperometric response of the ZnO nanoflower biosensor in different concentrations of glucose at 0.8 V in PBS solution (at pH = 7.0) [438].

Due to a SMOx’s high isoelectric point, glucose oxidase (GOx) with a low IEP of around 4.2 could be immobilized [437]. SMOx nanostructures and biopolymer composites also improved glucose biosensor activity. ZnO nanostructures have a high IEP (around 9.5), resulting in fast electron transfer rates and high enzyme loading activity [438,439,440]. An electrospun ZnO nanofiber glucose sensor exhibited a high and reproducible sensitivity of around 70.2 mAmM^−1^ cm^−2^ for glucose at 20–85 °C (Figure 26) [438].

**Figure 27:**
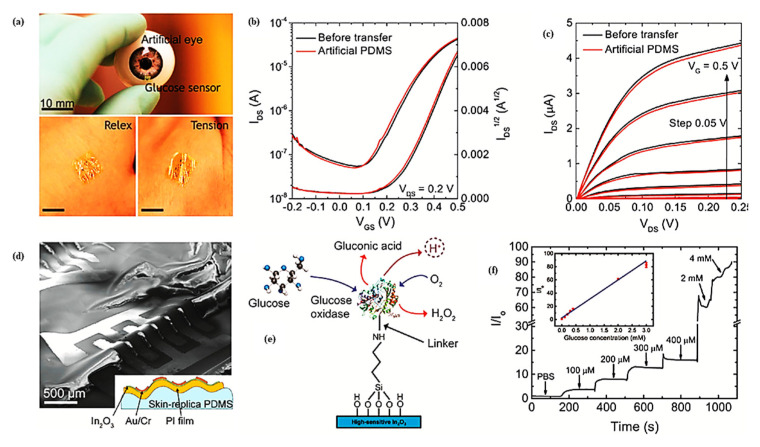
In_2_O_3_ FET− based biosensor. (**a**) Images of a contacted device on an artificial eye for glucose sensing in tears. Thin-film sensor contact with the skin during tension and relaxation; (**b**,**c**) device performance of thin-film In_2_O_3_ on a rigid substrate and flexible artificial PDMS skin, with the transfer of In_2_O_3_ FETs to replicas of skin under liquid gating with PBS solution at low voltage; (**d**) SEM image of In_2_O_3_ FET device on artificial PDMS skin replica; (**e**) enzymatic oxidation of D−glucose to generate gluconic acid and H_2_O_2_; (**f**) representation of In_2_O_3_ sensors for the concentration of D-glucose in a low range of human diabetic tears and high range of blood, with the standard deviation shown in the inset [448].

On a glassy carbon electrode (GCE), Fang et al. synthesized Nafion/GOx/ZnO hollow nanosphere composites. Since hollow nanosphere ZnO and GOx were adsorbed on the surface of the sensor [441,442], it exhibited high sensitivity (65.82 mA mM^−1^ cm^−2^) and fast response time (5 s). The authors reported direct electron transfer at a rate of 0.67 s^−1^ in glucose biosensors fabricated with GOx-immobilized ZnO/Cu nanocomposites [442]. There has been a surge in the development of wearable biosensors for the non-invasive monitoring of blood glucose. Field-effect transistors (FETs) based on SMOx gained attention in this area [443,444,445,446,447]. An electrochemical biosensor based on a FET coated with In_2_O_3_ 3.5 nm thick is shown in Figure 27. To monitor glucose levels in tears, this FET-based device was decorated with glucose oxidase [448]. Blood glucose concentrations are 70–180 mg/dL in a healthy individual, while tears contain 3–15 mg/dL glucose [448,449]. Through an ultra-thin In_2_O_3_ FET, the biosensor in Figure 27 could detect ultralow glucose concentrations in tears.

#### 6.1.2. Cholesterol-Oxidase-Immobilized Biosensors

**Figure 28:**
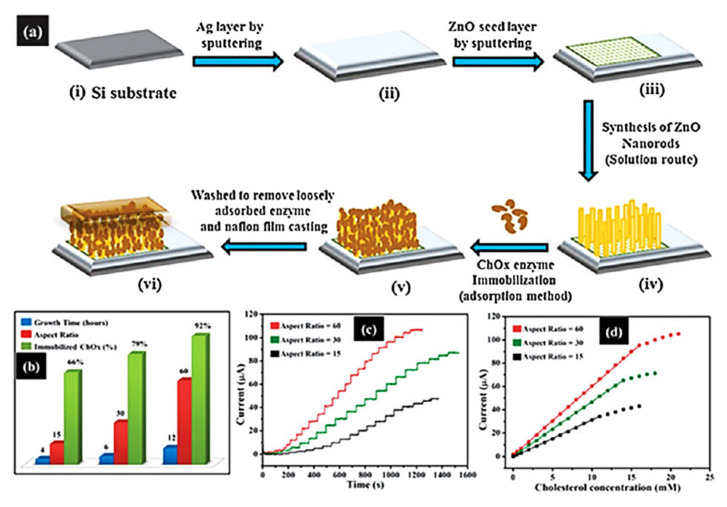
(**a**) Fabrication process flow for a cholesterol biosensor (i–vi); (**b**) relationship between the enzyme loading, the aspect ratio of ZnO nanorods, and growth time; (**c**) amperometric responses at an applied potential of +0.38 V for different aspect ratios of ZnO nanorods in the presence of cholesterol; (**d**) calibration curves of current response versus cholesterol concentration [456].

In order to fabricate an efficient and reliable cholesterol biosensor, SMOx was immobilized and stabilized with cholesterol oxidase (ChOx). This table outlines cholesterol biosensors constructed with different types of nanostructured modified electrodes [434,450]. At a low temperature, the Hahn group immobilized cholesterol oxidase on well-crystallized ZnO nanoflowers [434] and ZnO nanoparticles (NPs) [451]. It was shown that these biosensors had high reproducible sensitivity, a low detection limit, and a very fast response time (<5 s). ZnO nanofilms [452] and nano porous ZnO thin films [453] were also employed to fabricate cholesterol biosensors, which enhanced the electron transfer between cholesterol oxidase and electrodes. Composites, including platinum–gold-functionalized ZnO nanorods [454] and Pt-incorporated ZnO nanospheres [455], improved the sensitivity with low Km values. For the first time, Hahn et al. fabricated controlled ZnO nanorods directly on a silver electrode at 90 °C (Figure 28) [456]. The loaded cholesterol oxidase surface area of Malhotra and coworkers increased the electron transport between ChOx and the electrode, resulting in improved sensitivity [457]. SnO_2_ nanoparticles and chitosan composite films were developed by Ansari and co-workers for enhanced cholesterol adsorption [458]. A CH-SnO_2_/ITO nanocomposite was synthesized to improve the electrocatalytic activity and biocompatibility. SMOx-based highly-sensitive cholesterol biosensors were also developed using co-oxidase [459] and Fe_3_O_4_ nanoparticles [450].

#### 6.1.3. Urea- and Glutamate-Immobilized Biosensors

A urea biosensor based on ZnO nanowire arrays immobilized with urease (Urs) by Ali and co-workers had a sensitivity of about 52.8 mV per decade with linear response ranges (0.1–100 mM) [460]. Their bioelectrode also displayed a low response time (25 s), wide linear range (8 mM^−3^ mM), low detection limit (5.0 mM), and excellent stability [461]. Furthermore, Urs and glutamate dehydrogenase (GLDH) were combined to form a nanocomposite film on superparamagnetic Fe_3_O_4_ nanoparticles and chitosan that demonstrated a low Km value (0.56 mM) [462]. Nanostructured ZnO composites containing Urs and GLDH were reported to have a high sensitivity and a low detection limit of 13.5 mg dL^−1^, with a Km value of 6.1 mg dL^−1^ [463].

#### 6.1.4. Lipase-Immobilized Biosensors

It is also possible to form SMOx biosensors by immobilizing lipases. Solanki et al. describe a nanostructured cerium oxide film (35 nm) for lipase immobilization. With a linear range of 50–500 mg dL^−1^ and a detection limit of around 32.8 mg dL^−1^ at a low Km value (22.27 mg dL^−1^), the film exhibited a high affinity for tributyrin [464].

#### 6.1.5. Other Enzyme-Immobilized Biosensors

The TiO_2_ nanoneedle film immobilized with cytochrome complex (cyt c) described by Luo et al. facilitated electron transfer between redox enzymes and electrodes [465]. Their goal was to improve the enzyme activity against H_2_O_2_ released from human liver cancer cells. The detection of choline was achieved by electrochemically depositing MnO_2_ nanoparticles and nanowires, along with CH hydrogel and choline oxidase on GCE. MnO_2_ effectively trapped the target analyte on the electrode surface because of its large specific area. The linear detection range for α-MnO_2_ nanoparticles was 2.0–580 mM choline, while the linear detection range for β-MnO_2_ nanowires was 1.0–790 mM choline. In addition, an electro-chemiluminescence (ECL) lactate biosensor was made from nano-hybrids of ZnO-multiwalled carbon nanotubes (MWCNTs) modified with lactate oxidase and Nafion. Human blood plasma samples were tested using this ECL lactate biosensor.

### 6.2. Nucleic-Acid-Immobilized Biosensors

For DNA detection, SMOx materials are immobilized with nucleic acids (Table 4) [466,467,468,469,470]. Among the applications of these DNA biosensors are disease detection, genetic disorder screening, drug discovery, and forensics [471]. For the detection of acute promyelocytic leukemia, Zhang et al. immobilized single-stranded DNA sequences of 18-mer PML/RARA oligonucleotides on a carbon ionic liquid electrode modified with nanosized ZnO. The detection range was 1 × 10^−8^ to 1 × 10^−12^ M, with a detection limit of 2.5 × 10^−13^ M [467]. A ZnO-nanowire-based DNA biosensor made with multiwalled carbon nanotubes (MWCNTs) and gold nanoparticles was described by Wang et al. Sequence-specific target DNA can be detected using this biosensor. A single-stranded DNA probe with a thiol group at the end (HS-ssDNA) was covalently immobilized on the Au nanoparticle surface. A DNA biosensor based on [Ru(NH_3_)_6_]^3+^ as an intercalator [466] was capable of quantitatively detecting DNA in the range of 1.0 × 10^−13^ to 1.0 × 10^−7^ M. The 21-mer ssDNA of Mycobacterium tuberculosis was immobilized on a nano-ZrO_2_ film electrochemically deposited on a gold surface. With a detection limit of 65 mg mL^−1^ [472], this biosensor showed rapid diagnosis within 60 s. The biosensor was developed via a V_2_O_5_ nanobelt, MWCNTs, and chitosan nanocomposite material that was modified onto a carbon ionic liquid electrode (CILE) and immobilized with ssDNA for Yersinia enterocolitica detection. The biosensor detected complementary DNA at concentrations between 0.01 and 1.000 nM, with a detection limit of 1.76 pM [473].

### 6.3. Antibody-Immobilized Biosensors

Immunosensor sensitivity and stability were affected by the orientation, surface density, and antigen-binding efficiency of antibodies when immobilized onto functionalized surfaces. A model antibody–antigen system that represents the complex matrix immuno sensors encountered in reality was used to improve various surface functionalization processes and assess their effectiveness. Protein A/G enhanced antibody loading on surfaces substantially more than boronate ester chemistry. In spite of the fact that both enhance antigen binding by assisting in orientation-specific immobilization of antibodies, using protein A/G enhanced the antibody surface density, which is crucial to obtaining maximum antigen recognition [474,475].

**Figure 29:**
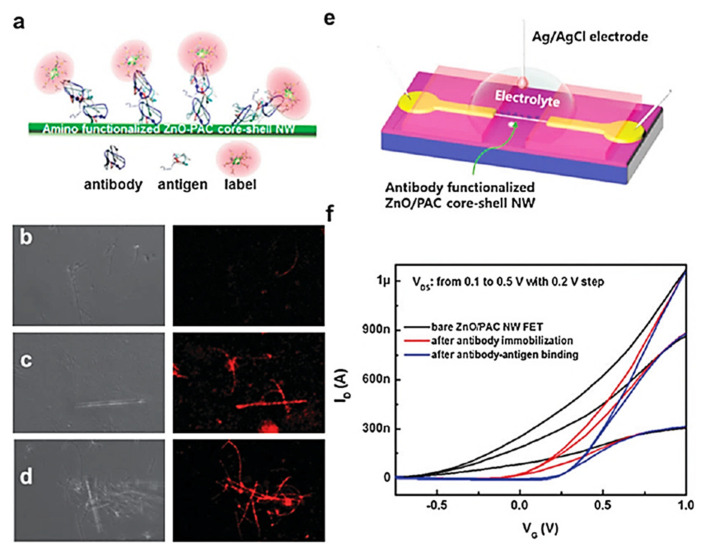
Electrical characterization of amino-functionalized ZnO/PAC nanowires for AFP detection. (**a**) Schematic view of anti−AFP immobilization via the sandwich binding method. The anti-AFP antibody was labeled with TRITC. Fluorescence microscopy images of an (**b**) untreated ZnO/PAC nanowire with AFP antigen, (**c**) an amino-functionalized ZnO/PAC nanowire with AFP antigen, and (**d**) an amino-functionalized ZnO/PAC nanowire with liver carcinoma. (**e**) Schematic view of an electrolyte-gated ZnO/PAC nanowire-based FET. (**f**) Current versus potential graph of a ZnO/PAC nanowire-based FET for sequential immobilizations [478].

In immunoassays, electrochemical immunosensors are used to detect antigens, antibodies, or other biochemical targets related to health issues, such as cancer antigens in serum and bacteria in food [476]. A CH-MnO_2_/MWNT-Ag composite impregnated with anti-AFP was electrodeposited on a CH-MnO_2_/MWNT-Ag composite developed by Che and co-workers [477]. Because of the high surface area and conductivity of the MWCNT-Ag, the detection range for AFP was 0.25–250 ng mL^−1^. Anti-AFP antibody immobilization on a ZnO/PAC nanowire FET allows for real-time, label-free detection of liver cancer markers (Figure 29) [478]. In addition, it was demonstrated that the biosensor could be used as a pH sensor. In an electrochemical immune sensor, Wei and his team utilized dumbbell-like Au–Fe_3_O_4_ nanoparticles for detecting prostate-specific antigen (PSA), which is a cancer biomarker. PSA detection was achieved by immobilizing primary anti-PSA antibodies on graphene and secondary antibodies on Au–Fe_3_O_4_ nanoparticles [479]. The immune sensor has a wide linear range of detection (0.01–10 ng mL^−1^), a low detection limit (5 pg mL^−1^), and excellent reproducibility and stability.

### 6.4. Other Biomaterial-Immobilized Biosensors

Graham and coworkers demonstrated the fabrication of a SMOx-based bioelectrode for on-chip, long-term, and noninvasive cell culture assays and label-free high-content screening. In addition to detecting the fast electrical activity of neurons, the bioelectrode detected slow changes in impedance as the cells grew and divided. The team showed that a silver- and TiO_2_-based biosensor could be used to analyze prokaryotic gene expression in real time [480] Table 4 compares SMOx-based biosensors.

**Table 4:** SMOx-based immobilized biosensors.

Target Biomolecule	Electrode	Sensitivity	Detection Limit (mM)	Linear Range (mM)	Response Time (s)/ Potential (V)	Ref.
**Glucose**	GCE/ZnO NF/PVA/GOx/L-Cys	70.2 mA mM^−1^ cm^−2^	1	0.25–19	<4/+0.80	[438]
	GCE/ZnO-HNSPs/GOx/Nafion	65.82 mA mM^−1^ cm^−2^	1	0.005–13.15	<5/+0.8	[441]
	GCE/ZnO NRs/GOx/CHIT	25.7 mA mM^−1^ cm^−2^	10	0.01–0.25/0.3–0.7	<2/+0.8	[481]
	Au/ZnO NT/GOx/Nafion	21.7 mA mM^−1^ cm^−2^	1	0.05–12.0	3/+0.8	[482]
	Au/ZnO nano-tetrapods/GOx/Nafion	25.3 mA mM^−1^ cm^−2^	4	0.005–6.5	<6/+0.8	[483]
	ITO/ZnO NT arrays/GOx/Nafion	30.85 mA mM^−1^ cm^−2^	10	0.01–4.2	<6/+0.80	[484]
	PET/Au/ZnO-NWs/GOx/Nafion	19.5 mA mM^−1^ cm^−2^	<50	0.2–2.0	<5/+0.80	[485]
	PDDA/GOx/ZnO/MWNTs	50.2 mA mM^−1^ cm^−2^	0.25	0.1–16	–/–	[486]
	ITO/Cu/ZnO/HRP-GOx/Con A/CS-Au	0.097 mA mM^−1^ cm^−2^	40	1.0–15.0	<6/−0.39	[442]
	GCE/porous TiO_2_/GOx/Nafion	0.3 mA mM^−1^ cm^−2^	-	0.15–1.2	<10/−0.45	[487]
	GCE/TiO_2_-GR/GOx	6.2 mA mM^−1^ cm^−2^	-	0–8.0	–/−0.60	[488]
	Au/CuO/GOx/Nafion	47.19 mA mM^−1^ cm^−2^	1.37	0.01–10.0	<5/-	[489]
	ITO/CeO_2_ NRs/GOx	0.165 mA mM^−1^ cm^−2^	100	2.0–26.0	1–2/+0.80	[490]
	Pt/GOx/Fe_3_O_4_/Chitosan/Nafion	11.54 mA mM^−1^ cm^−2^	6	0.006–2.2	–/–	[491]
	Au/MgO/GOx/Nafion	31.6 mA mM^−1^ cm^−2^	0.068	0.001–0.009	<5/	[492]
	Pt/NiO doped ZnONRs/GOx	61.78 mA mM^−1^ cm^−2^	2.5	0.5–8.0	<5/+0.39	[439]
	GCE/NiO/GOx/CHIT	3.43 mA mM^−1^ cm^−2^	47	1.5–7	<8/+0.	[440]
**Cholesterol**	Au/flower-shaped ZnO/ChOx/Nafion	61.7 mA mM^−1^ cm^2^	0.012	1.0–15.0	<5/-	[434]
	Au/ZnO NPs/ChOx/Nafion	23.7 mA mM^−1^ cm^2^	0.00037	0.001–0.5	<5/+0.355	[493]
	Ag/ZnO/ChOx	35.2 mV per decade	-	0.001–10.0	–/–	[494]
	ITO/NS-CeO_2_/ChOx	2 mA mg dL^−1^ cm^2^	-	0.26–10.36	~15/+0.50	[495]
	ITO/CH-SnO_2_/ChOx	34.7 mA mg dL^−1^ cm^2^	130	0.26–10.36	0.5/-	[458]
	ITO/NanoFe_3_O_4_/ChOx	86 Ω mg^−1^ dL cm^−2^	6.5	0.0065–10.36	25/+0.06	[450]
**Nucleic acid**	ssDNA/ZnO/MWNTs/ CHIT/GCE	-	2.8 × 10^−12^ mol L^−1^	1.0 × 10^−11^ − 1.0 × 10^−6^ mol L^−1^	-	[496]
	ssDNA/AuNPs/MWNTs/ ZnO NWs/GCE	-	3.5 × 10^−14^ M	1.0 × 10^−13^ − 1.0 × 10^−7^ M	-	[466]
	ssDNA/ZnO/CILE	-	2.5 × 10^−13^ mol L^−1^	1.0 × 10^−12^ − 1.0 × 10^−8^ mol L^−1^	-	[467]
	ssDNA/Cu_2_O/CPE	-	1.0 × 10^−10^ mol L^−1^	1.0 × 10^−10^ − 1 × 10^−6^ mol L^−1^	-	[469]
	ssDNA/CeO_2_-SWNTs BMIMPF6/GCE	-	2.3 × 10^−13^ mol L^−1^	1.0 × 10^−12^ − 1.0 × 10^−7^ mol L^−1^	-	[497]
	ssCT-DNA/CH-Fe_3_O_4_/ITO	-	0.0025 ppm	1–300 ppm	-	[462]
	PNA/Fe_3_O_4_-GOPS/ITO	-	0.1 × 10^−15^ M	0.1 × 10^−15^ − 50.0 × 10^−15^ M	-	[470]
**Antibody**	BSA/anti-AFP/CH-MnO_2_/ MWNT-Ag/GCE	-	0.08 ng mL^−1^	0.25–250 ng mL^−1^	-	[477]
	BSA/r-IgGs/Nano-ZnO/ITO	189 Ω nM^−1^ dm^−3^ cm^−2^	0.006 nM dm^−3^	0.006–0.01 nM dm^−3^	-	[498]
	Anti-CEA/Fe_3_O_4_ NRs/CPE	-	0.9 ng mL^−1^	1.5–80 ng mL^−1^	-	[499]
	HRP-anti-hIgGAu/SiO_2_ NPs PTHGCE	-	0.035 ng mL^−1^	0.1–200 ng mL^−1^	-	[500]

### 6.5. Non-Enzymatic Biosensors

The direct electrochemistry of glucose (oxidation or reduction) was used for non-enzymatic glucose sensing, which was quick and inexpensive [501]. The direct oxidation of glucose using noble metal electrodes, however, has three significant drawbacks [502,503]: (1) limited glucose sensitivity due to the slow electrooxidation kinetics of glucose with conventional electrodes, (2) low selectivity because several sugars can be oxidized in the same potential range as glucose, and (3) decreased electrode activity due to ion contamination. An increased electrode surface area enabled more glucose to come into contact with the electrode surface, thus eliminating the sensitivity and selectivity limitations. In the non-enzymatic process of glucose oxidation, hydrogen atoms are abstracted concurrently with organic species adsorption [504]. This is the rate-determining step in the glucose electrooxidation catalytic process. IHOAM is a hypothesis put forth by Bruke et al. to explain the intricate electrocatalytic process of glucose [504].

Metals, particularly noble metals, were investigated as electrode materials for non-enzymatic glucose biosensors [501,502]. Several metal alloys and hybrid materials have been developed as a result of advances in materials science in order to improve the properties of noble metals and metal oxides alone. Xiao et al. developed a flexible electrochemical glucose sensor by incorporating the nanocomposite PtAu alloy and MnO_2_ into graphene paper [505]. As a result of electrodeposition on graphene paper, a PtAu-MnO_2_ nanocomposite was developed with tight contact between the PtAu alloy and the MnO_2_. This glucose sensor had a linear range of 0.1 mM to 30 mM and a high sensitivity of 58.54 μA mM^−1^ cm^−2^ [505]. Lee et al. created a disposable non-enzymatic blood glucose sensor strip using microporous Pt as an electrode material and poly(vinyl acetate) as a binding material. The mixture was then applied to a polyimide film surface with a conducting circuit screen printed on it. In whole human blood, the sensor showed acceptable stabilization for 30 days with a sensitivity of 0.0054 μA cm^−2^ mgdL^−1^ [506]. An electrode fabricated from nanoporous Cu (NPC) was used by Chen et al. to make a portable micro glucose sensor [507]. In this non-enzymatic sensor, CuO nanocoral arrays had high conductivity and high glucose catalytic activity. It had a linear range of 0.0005 to 5 mM and a sensitivity of 1621 μA mM^−1^ cm^−2^. Liu et al. combined a wet chemical process with an annealing procedure to create 3D copper oxide nanowire arrays (CuONWAs). In the end, the CuONWA/CF platform served as a glucose sensor [508]. Because the copper foam and nanowire arrays increase the surface area of the device, its sensitivity is improved. By using tellurium microtubes, Guascito et al. altered the surface of a Pt electrode using a drop-casting technique. It was found that the non-enzymatic glucose sensor was more sensitive, stable, and reproducible when compared with a Pt electrode that had not been modified [509].

## 7. Conclusions

A SMOx-based sensor translates a response into an electrical signal by using a receptor-transducer device. A wide range of applications, such as the detection of diseases and illnesses, environmental monitoring, water and food quality monitoring, and drug delivery, prompted scientists and researchers to develop more sensitive and selective sensors. SMOx-based sensors need to capture recognition signals efficiently and convert them into electrochemical, electrical, optical, gravimetric, or acoustic signals (transduction process). Increasing transducer performance is another challenge, as it allows for increased sensitivity, faster response times, reproducibility, and lowering detection limits, even to detect single molecules and miniaturization of the sensing devices. Combining sensing technology with nano-SMOx-based devices, like zero- to three-dimensional FETs and IoT, with high surface-to-volume ratios, good conductivities, shock-bearing properties, and colour tuning can overcome these challenges. In this review, we provide an overview of the development of SMOx-based sensors.

In this review, we discussed, the recent advancements in semiconductor metal oxides (SMOx) for gas sensing, chemical sensing, and biosensing applications. The unique intrinsic chemical, physical, optical, and electronic properties of SMOxs entail low detection limits, high sensitivity, and fast response time, making SMOx materials a popular choice for sensing applications. Over the past few decades, the synthesis of SMOx-based nanomaterials in varying sizes, structures, and crystal morphologies has enabled the detection of gases, such as H_2_, CO, O_2_, SO_2_, NO_2_, and H_2_S, and various chemicals and biomolecules like glucose, cholesterol, nucleic acids, and other important biomolecules. SMOx materials possess a large specific surface area, superior electron transport rate, extraordinary permeability, and active reaction sites, making them well-suited for stable sensing of specific gases, chemicals, and biomolecules at room temperature. Different architectural morphologies of nano-SMOxs are used to accelerate the rate of electron transport, resulting in improved sensitivity.

This review also highlights the improvement of sensing properties through various strategies, such as loading with nanomaterials, doping with elements, and constructing heterojunctions with other functional materials. The sensing properties of SMOx-based nanoparticles were found to be extensively improved by decorating their surfaces with nanomaterials, which change electron accumulation and enhance their catalytic effect through electronic and chemical excitation, respectively. The incorporation of a large number of dopants into the lattice of SMOx changes their crystal and electronic structure, reducing the bandwidth and increasing the active sites on the surface, which affects the sensitivity and selectivity. The formation of heterojunctions effectively rectifies electron transfer on the surface between two materials, resulting in improved sensitivity. The composites of SMOx with other functional materials also enhance the sensitivity and response rate at low temperatures due to possible synergistic effects and defect structures. These developments in SMOx composites have the potential to enable their practical applications.

## 8. Future Aspects

Despite the significant advancements in the development of semiconductor metal oxide (SMOx)-based sensors, there is still room for improvement in terms of selectivity, sensitivity, and working temperature. Recent studies highlighted the potential use of nanostructured SMOx-based sensors for their applications in sensing fields, specifically as gas, chemical, and biological sensors. However, new materials and heterostructure designs require special attention for further development mechanisms, including influencing the sensing activity. The future of SMOx-based sensor research can focus on (1) developing new materials and new heterojunction interfaces and (2) exploring the mechanisms that influence sensing activity and improving selectivity and sensitivity for the detection of gas, chemical, and biological molecules. The implementation of new strategies, such as doping with electronically active materials, functionalization of SMOxs with unique functional groups, and development of sensing materials, is crucial in fabricating more sensitive novel sensors. The use of SMOx-based sensors was found to offer several advantages over traditional sensors, including higher sensitivity, exceptional selectivity, quick response time, low detection limits, and compact size. The objective of this review was to aid in the development of a new generation of sensing tools that can identify a wide variety of molecules in a variety of contexts, such as environmental danger gases and therapeutic biomolecules. Rapid and precise detection of gases, chemicals, and biological materials is crucial for an effective response. Therefore, to achieve this goal, a hybrid sensor system might be highly desirable. Moreover, integrated sensors for the detection of hybridized gas; chemicals; a mixture of toxic gases; and hazardous biological substances, like pathogenic microorganisms, bacteria, and viruses, can be realized with SMOx materials. These unique SMOx-based sensors can be utilized in a wide range of applications, such as in hospitals, defense areas, or war zones; for pharmaceutical, pesticide, textile, and meat industries; and in houses. These sensors also detect the growing threat of natural infectious diseases or industrial accidents. In summary, SMOx-based sensors have the potential to uncover new opportunities for the detection, identification, and quantification of toxic gases in various settings, such as food, hospitals, and the ocean. These new and innovative approaches must be highly selective, sensitive, reliable, fast-responding, and capable of autonomous screening. Moreover, they should be able to transmit information securely and wirelessly in real time. Also, SMOxs can be used for different types of optical sensor fabrications, including fiber optics and waveguide-based sensors, and have a lot of advantages over other types of sensors [508].

## Figures and Tables

**Table 1:** Some semiconductor metal oxide (SMOx)-based sensors with their structural parameters.

SMOx	Lattice Parameters	Applications	References
**Nickel oxide (NiO)**	**Cubic**	1. NO_2,_ CO gas sensing	[58,59,60,61,62,63,64,65]
		2. Ammonia sensing	
	Fm3m	3. Ethanol sensing	
	a = 2.983 Å	4. Uric acid sensing	
	b = 2.983 Å	5. Lactic acid sensing	
	c = 5.160 Å	6. Glucose sensing	
**Cobalt oxide (CoO)**	**Cubic**	1. Gas sensing	[66,67,68,69]
		2. Oxygen sensing	
	Fm3m	3. Aceton sensing	
	a = 3.024 Å		
	b = 3.012 Å		
	c = 5.316 Å		
	**Hexagonal**		
	P6_3_mc		
	a = 3.269 Å		
	b = 5.289 Å		
	c = 5.646 Å		
**Tin dioxide (SnO_2_)**	**Cubic**	1. Gas sensors	[70,71,72,73,74,75,76,77,78,79,80,81]
		2. Formaldehyde sensing	
	Fm3m	3. H_2_S sensing	
	a = 3.640 Å	4. Alkene sensing	
	b = 3.640 Å	5. H_2_ sensing	
	c = 3.640 Å	6. Biomarker of lung cancer	
		7. CO sensing	
	**Tetragonal**		
	P4/mnm		
	a = 4.832 Å		
	b = 4.832 Å		
	c = 3.243 Å		
**Titanium dioxide (TiO_2_)**	**Tetragonal**	1. Hazardous gas sensing	[82,83,84,85,86,87,88,89,90]
		2. Gas and UV sensor	
	I4_1_/amd	3. Phosphopeptide sensing	
	a = 5.566 Å	4. Chemical sensing	
	b = 5.566 Å	5. Lactate sensing	
	c = 5.566 Å	6. Biosensors	
	**Tetragonal**		
	P4_2_/mnm		
	a = 4.653 Å		
	b = 4.653 Å		
	c = 2.969 Å		
**Zinc oxide (ZnO)**	**Cubic**	1. Gas sensor	[91,92,93,94,95,96,97,98]
		2. H_2_ sensing	
	Fm3m	3. Chemical sensing	
	a = 3.068 Å	4. Pesticide detection	
	b = 3.068 Å	5. Biosensors	
	c = 3.068 Å		
	**Hexagonal**		
	P6_3_mc		
	a = 3.289 Å		
	b = 3.289 Å		
	c = 5.307 Å		
**Trimanganese tetraoxide (Mn_3_O_4_)**	**Tetragonal**	1. H_2_ gas sensing	[99,100]
		2. Nitrogen sensing	
	I41/amd		
	a = 5.870 Å		
	b = 6.348 Å		
	c = 5.873 Å		

**Table 2:** Different gas-sensing activities of SMOx-based nanomaterials.

Analyte Gas	Layer Composition	Meas. Temp.	Concentration	Response	t_res_	t_rec_	Selective Agents	Ref.
**CO**	40% In_2_O_3_-SnO_2_	250 °C	1000 ppm	16	NA	NA	NA	[322]
	SnO_2_@In_2_O_3_	300 °C	200 ppm	1.9	135 s	460 s	NA	[323]
	SnO_2_@NiO	250 °C	500 ppm	15.9	NA	NA	CH_4_	[324]
	50 wt% Co_3_O_4_-SnO_2_	100 °C	1000 ppm	175	NA	NA	H_2_	[325]
	(3 wt% ZnO-SnO_2_)@ CuO	235 °C	200 ppm	13.4	NA	NA	H_2_	[326]
	20 wt% WO_3_-MoO_3_	200 °C	15 ppm	300	2 min	2 min	NA	[248]
**H_2_**	(3 wt% ZnO-SnO_2_)@CuO	305 °C	200 ppm	16	NA	NA	CO	[326]
	SnO_2_@2.6mol% ZnO	350 °C	100 ppm	18.4	NA	NA	CO, NH_3_, CH_4_	[327]
	(0.005 mol MoO_3_)-SnO_2_	240 °C	1000 ppm	10	5 s	10 s	NA	[328]
	1 wt% Co_3_O_4_-SnO_2_	250 °C	1000 ppm	9100	NA	NA	CO	[325]
	TiO_2_/NiO	200 °C	10,000 ppm	70	NA	NA	NA	[329]
	ZnO@SnO_2_	400 °C	500 ppm	70	NA	NA	NA	[330]
**NO_2_**	SnO_2_@ZnO	RT	5 ppm	0.4	~50 s	~450 s	NA	[331]
	40% ZnO-SnO_2_	250 °C	500 ppm	34.5	NA	NA	NA	[332]
	20% WO_2_-SnO_2_	200 °C	200 ppm	186	NA	NA	NA	[333]
	40% In_2_O_3_-SnO_2_	200 °C	1000 ppm	7.5	NA	NA	NA	[322]
	5% Eu_2_O_3_-ZnO	300 °C	3 ppm	16	3 min	3 min	CO	[334]
**H_2_S**	Cu_2_O/SnO_2_	RT	50 ppm	45	NA	NA	Toluene, LPG	[335]
	6% CuO/SnO_2_	150 °C	20 ppm	4300	3 s	NA	NA	[336]
	1 mol% CeO_2_-SnO_2_	300 °K	5 ppm	3	40 s	20 s	LPG, EtOH, NO_x_, CO	[337]
	SnO_2_@ZnO	350 °C	500 ppm	2.1	NA	NA	CO, CH_4_	[324]
	(5 wt% ZnO-SnO_2_)@3.68 wt% CuO	150 °C	50 ppm	60,443	15 s	7 min	NO_x_, LPG, CO_2_, CH_4_	[338]
	SnO_2_	350 °C	50 ppm	10	NA	NA	NA	[338]
	ZnO	100 °C	100 ppm	25	NA	NA	NH_3_, MeOH, EtOH,butanol, acetone, ether	[339]
**NH_3_**	ZnO@Cr_2_O_3_	RT	300 ppm	13.7	25 s	75 s	LPG, CO_2_, EtOH, H_2_, Cl_2_	[299]
	2mol%α-Fe_2_O_3_-ZnO	RT	0.4 ppm	10,000	20 s	20 s	TMA, EtOH, MeOH	[340]
**Ethanol**	ZnSnO_3_@SnO_2_	270 °C	50 ppm	27.8	1 s	1.8 s	Acetone, benzene, chloroform, MeOH, formaldehyde, CO	[341]
	5 wt% La_2_O_3_-SnO_2_	300 °C	1000 ppm	740	20 min	20 min	NA	[318]
	50 wt% SnO_2_-ZnO	300 °C	200 ppm	4.69	72 s	NA	Acetone, CO, H_2_, NO_2_, C_3_H_8_	[342]
	1.5 mol% Fe_2_O_3_-SnO_2_	250 °C	10 ppm	24	NA	NA	NA	[343]
	ZnO	300 °C	100 ppm	7	NA	NA	NA	[344]
	ZnO@ZnS	210 °C	1000 ppm	23	15 s	15 s	NA	[345]
	ZnO@ZnS @Graphene	210 °C	1000 ppm	38	15 s	15 s	Acetone, formaldehyde, benzene, cyclohexane	[345]
	25 wt% SnO_2_-ZnO	350 °C	100 ppb	~82	NA	NA	NA	[311]
	2:8 mol CuO: ZnO	115 °C	100 ppm	96	13 s	5 s	NA	[346]
	20 wt% SnO_2_-TiO_2_	553 °K	200 ppm	51	10–15 s	14–20 s	NA	[347]
	ZnO-Co_3_O_4_	170 °C	100 ppm	46	NA	NA	NA	[348]
**Ethylene**	0.3 wt% WO_3_-SnO_2_	300 °C	6 ppm	1.7	~10 min	~10 min	NA	[349]
**O_3_**	MoO_3_-TiO_2_	300 °C	100 ppb	1.7	20 s	2 min	NA	[350]
**SO_2_**	1 mol% NiO-SnO_2_	25 °C	18 ppm	0.84	4.5 min	15 min	O_2_, C_3_H_8_, NO_x_	[351]
**LPG**	ZnO@0.47 wt% Cr_2_O_3_	350 °C	100 ppm	46	18 s	42 s	NH_3_, CO_2_, EtOH, H_2_,	[352]
**TMA**	10 wt% ZnO-SnO_2_	330 °C	50 ppm	126	2 s	5 s	NH_3_, DMA, MA, EtOH, MeOH, Acetone	[353]

**Table 3:** Different SMOx-based chemical sensors.

SMOx Composite Materials	Analyte Gas	Detection Limit (Conc.)	Senor Response/Temperature	Response/Recovery Time	References
**SnO_2_ nanowire**	H_2_	10 ppm	~0.4/300 °C	N/A	[358]
**SnO_2_ hollow sphere**	CO	50 ppm	-/300–350 °C	<1 min./30 min.	[360]
**SnO_2_ nanocrystals**	NO_2_	100 ppb	-/300 °C	N/A	[361]
**SnO_2_ porous NPs**	H_2_/CO	160/200 ppm	-/300 °C	NA	[362]
**SnO_2_ flower-like**	CO	50 ppm	~2.13/350 °C	26 s/34 s	[362]
**SnO_2_ nanorods**	H_2_	100 ppm	~13/150 °C	N/A	[386]
**Plasma-modified SnO_2_ nanowire**	Ethanol	100 ppm	-/250 °C	N/A	[366]
**Pt@SnO_2_ nanorods**	Ethanol	10 ppm	3.7/300 °C	2 s/20 s	[367]
**NiO-SnO_2_ nanofibers**	Ethanol	100 ppm	25.5/300 °C	2 s/3 s	[387]
**p-NiO/n-SnO_2_ heterojunction composite nanofibers**	H_2_	100 ppm	13.6/320 °C	~3 S/~3 S	[388]
**ZnO nanorods**	Ethanol	1 ppb	~10/300 °C	100 s/-	[389]
**ZnO nanorod arrays**	H_2_	500 ppm	-/250 °C	6 min/17 min	[390,391]
**ZnO nanowire**	NO_2_	0.5 ppm	-/225 °C	N/A	[392]
**Pt adsorbed single crystalline ZnO nanowires**	NH_3_	1000 ppm	-/350 °C	100 s/100 s	[393]
**Co-doped ZnO nanorods**	CO	50 ppm	-/350 °C	N/A	[372]
**Pd nanodots–functionalized ZnO nanowire**	CO	100 ppb	1.02/20 °C	120 s/180 s	[373]
**Ga_2_O_3_ nanowire**	O_2_	1% O2	4.75/300 °C	N/A	[394]
**NiO nanotubes**	Ethanol	200 ppm	22.6/250 °C	N/A	[395]
**α** **-Fe_2_O_3_ hollow spheres**	Ethanol	10 ppm	~5/RT	N/A	[396]
**Fe_2_TiO_5_/** **α** **-Fe_2_O_3_ nanocomposite**	Ethanol	10 ppm	~10/320 °C	28 s/21 s	[397]
**Fe_2_O_3_–TiO_2_ tube-like nanostructures**	Ethanol	500 ppm	8.2/270 °C	N/A	[377]
**In_2_O_3_ nanofibers**	Ethanol	100 ppm	~14/300 °C	1 s/5 s	[378]
**In_2_O_3_ nanoparticles**	NO_x_	200 ppm	~10,000/150 °C	N/A	[379]
**Sn-doped In_2_O_3_ nanopowders**	CO	50 ppm	4/250 °C	N/A	[380]
**In_2_O_3_ hollow microspheres**	Ethanol	100 ppm	137.2/400 °C	N/A	[381]
**Ag-doped In_2_O_3_ nanoparticles**	Alcohol vapors	100 ppm	-/150 °C	42 s/34 s	[383]
**In_2_O_3_ nanorods**	Formaldehyde	32 ppm	-/300 °C	276 s/65 s	[384]
**Mesoporous In_2_O_3_ nanorods**	Ethanol	500 ppb	1.71/290 °C	6 s/8 s	[385]
**Pt/In_2_O_3_ nanofibers**	H_2_S	600 ppm	1490/200 °C	60 s/120 s	[398]
**CuO nanoribbons**	Methanol	5 ppm	~1.4/RT	2–4 s/3–7 s	[399]
**Porous CuO nanowire**	H_2_	6% H_2_	407%/250 °C	72 s/156 s	[400]
**Co_3_O_4_ hollow spheres**	Butanol	10 ppm	3/100 °C	1–3 s/4–8 s	[401]
**WO_3_ nanowires**	H_2_S	1 ppm	48/250 °C	-	[273]
**WO_3_ nanoplates**	Ethanol	10 ppm	~1.9/300 °C	-	[402]

## Data Availability

Not applicable.
